# Passive Magnetic-Flux-Concentrator Based Electromagnetic Targeting System for Endobronchoscopy

**DOI:** 10.3390/s19235105

**Published:** 2019-11-21

**Authors:** Chin-Chung Chen, Ching-Kai Lin, Chen-Wei Chang, Yun-Chien Cheng, Jia-En Chen, Sung-Lin Tsai, Tien-Kan Chung

**Affiliations:** 1 Department of Mechanical Engineering, National Chiao Tung University, Hsinchu 30010, Taiwan; chinchung.me02g@nctu.edu.tw (C.-C.C.); vanhalen@ntuh.gov.tw (C.-K.L.); wychang.me05g@g2.nctu.edu.tw (C.-W.C.); yccheng@nctu.edu.tw (Y.-C.C.); kh14760.me06g@nctu.edu.tw (J.-E.C.); peter.me03@g2.nctu.edu.tw (S.-L.T.); 2 Department of Internal Medicine, National Taiwan University Hospital, Taipei 10002, Taiwan; 3 Department of Internal Medicine, National Taiwan University Hospital Hsin-Chu Branch, Hsinchu 30059, Taiwan; 4 Department of Medicine, National Taiwan University Cancer Center, Taipei 10672, Taiwan; 5 International College of Semiconductor Technology, National Chiao Tung University, Hsinchu 30010, Taiwan

**Keywords:** passive magnetic-flux concentrator, magnetic, electromagnetic, targeting, tracking, locating, endobronchoscope, endoscope, lung cancer tumor/lesion

## Abstract

In this paper, we demonstrate an innovative electromagnetic targeting system utilizing a passive magnetic-flux-concentrator for tracking endobronchoscope used in the diagnosis process of lung cancer tumors/lesions. The system consists of a magnetic-flux emitting coil, a magnetic-flux receiving electromagnets-array, and high permeability silicon-steel sheets rolled as a collar (as the passive magnetic-flux-concentrator) fixed in a guide sheath of an endobronchoscope. The emitting coil is used to produce AC magnetic-flux, which is consequently received by the receiving electromagnets-array. Due to the electromagnetic-induction, a voltage is induced in the receiving electromagnets-array. When the endobronchoscope’s guide sheath (with the silicon-steel collar) travels between the emitting coil and the receiving electromagnets-arrays, the magnetic flux is concentrated by the silicon-steel collar and thereby the induced voltage is changed. Through analyzing the voltage–pattern change, the location of the silicon–steel collar with the guide sheath is targeted. For testing, a bronchial-tree model for training medical doctors and operators is used to test our system. According to experimental results, the system is successfully verified to be able to target the endobronchoscope in the bronchial-tree model. The targeting errors on the *x*-, *y*- and *z*-axes are 9 mm, 10 mm, and 5 mm, respectively.

## 1. Introduction

To date, endobronchoscopy, a minimal invasive method, has been widely applied to diagnose lung cancer tumors/lesions. The endobronchoscope-based diagnosis procedure is much less complicated than other diagnostic tools such as computer–tomography (CT) guided biopsy and thoracotomy/surgical-based biopsy [1]. Among the enbronchoscope-based diagnosis procedures, endobronchial ultrasound (EBUS) is a useful, powerful, and safe tool for confirming the location of lung tumor/lesion in endobronchial routes [2,3]. Furthermore, by combining the standard EBUS with advanced endobronchoscopic navigation/targeting technology, we can obtain higher diagnostic yield and shorter procedure time for lung cancer tumor/lesion diagnosis [4]. The detailed procedure of operating the standard EBUS combined with the navigation/targeting technology is described in the following. Before the procedure, every patient must take a thin-slide CT scan of his/her lung. After the scan, the CT image is imported to an image processing system to reconstruct the 3D image of the bronchial tree of the patient and mark/register potential tumors’/lesions’ location. Subsequently, the EBUS radial probe was inserted into a guide sheath and then aligned distal-ends of the probe and the sheath by referencing alignment marks. After this, the navigation/targeting procedure starts. Medical doctors and operators insert the EBUS radial probe with the guide sheath from the oral or nasal route to bronchus. After insertion, by using the navigation/targeting system, the medical doctors and operators navigate the probe with the sheath to the most reachable distal parts of the bronchus that have tumors/lesions and subsequently find/locate the tumors/lesion by referencing the EBUS image. Once the target tumor/lesion is found, the medical doctors and operators remove the EBUS radial probe from the guide sheath and subsequently insert the biopsy forceps into the guide sheath to collect the tissue specimens of the tumors/lesions. The tissue specimens are placed in formalin for histological examination. However, within the same navigation/targeting/EBUS-localization/biopsy procedure for the same tumor/lesion, medical doctors and operators have to alternatively perform navigation/targeting and EBUS localization many times (note: cannot be performed simultaneously due to EBUS principles). Therefore, performing accurate and complete navigation/targeting with EBUS to locate/target tumors/lesions is really a very time-consuming process. Due to this, researchers are still searching for an accurate, complete, and time-saving navigation/targeting approach for an endobronchoscope diagnosis procedure to locate/target tumors/lesions.

Recently, magnetic sensors [5,6,7,8,9,10,11,12,13,14,15] are developed for electromagnetic and magnetic navigating/targeting/tracking systems which become important alternative navigating/targeting/tracking approaches for surgical/clinical applications [16,17,18,19,20,21,22,23,24]. More recently, these magnetic sensor based electromagnetic navigations are integrated with standard EBUS as a hybrid system to perform a more accurate navigating/targeting endobronchoscope to target/locate lung tumors/lesions [25,26,27,28,29,30]. The system consists of a magnetic sensor attached on a distal-end of an EBUS convex probe (whose diameter is much bigger than a radial probe), a magnetic-sensors-module attached to the patient as a location reference, a huge magnetic-field generator, and an image/signal-processing module. The targeting principle is described as the following. For official diagnosis protocol using electromagnetic/EBUS hybrid targeting system, the above standard preparation process (i.e., CT scanning, bronchial tree image reconstructing, and potential lung tumors’/lesions’ location marking/registering) must be conducted before the navigating/targeting/tracking. Note: however, in this paper, because we only focus on develop a novel targeting process, the above preparation process is unnecessary and thus can be skipped. When the targeting starts, the operator inserts an EBUS convex probe (with a magnetic sensor fixed on the tip of the probe) into the patient’s airway and then to a bronchial-tree. The magnetic field generator produces a magnetic field across the bronchus to the magnetic sensor. Due to this, the magnetic sensor produces electrical signals and consequently transmits the signals to the signal-processing system. Through signal processing, the location of the sensor/tip-of-probe is real-time targeted. After the tumor/lesion is located/targeted by using the EBUS probe, a dedicated puncture needle (similar function as biopsy forceps) is inserted to collect the tissue specimens of the tumor/lesion. However, this hybrid targeting approach is only able to real-time target the EBUS convex probe (which is generally used to diagnose mediastinal tumors/lesions). The approach has not been used (or may not be able) to real-time target a EBUS radial probe which is specifically used to diagnose peripheral lung tumors/lesions (Note: because the diameter of the radial probe is much smaller than that of the convex probe, the radial probe is used specifically to target peripheral lung tumors/lesions (while the convex probe is used generally to target mediastinal tumors/lesions). In early-stage (small-tumor/lesion) diagnosis, peripheral lung tumors/lesions are more critical than mediastinal tumors/lesions). Most importantly, the tip of the probe of the system is fixed with a magnetic sensor which is an active sensing component, and thus requires meters-long power and signal wires connected from outside of the endoscope to the inside of the probe (through a tiny working channel in the probe). This causes a complex wire-routing/deploying problem. In addition, the probe (with a magnetic sensor fixed on the probe’s tip) is very expensive, complicated, and non-compatible to other system. Furthermore, the electromagnetic navigation/targeting system corresponding to the probe is also very expensive and complicated. Due to these, a special sensing element which owns passive, powerless, and wireless features is needed to replace the active magnetic sensor on the tip of the probe. In addition, a new, cheap, easy, and compatible radial probe and corresponding electromagnetic targeting approach are also needed. Furthermore, a demonstration of combining this EBUS radial probe based electromagnetic targeting approach for real-time navigating/targeting diagnosis for peripheral lung tumors/lesions is also needed.

Recently, we reported electromagnetic-induction-based methods for targeting/locating distal screw-hole in intramedullary interlocking-nail surgery [31,32,33,34,35]. The targeting method consists of a c-shaped electromagnet (which contains an emitting coil, a pick-up coil, and a c-shape silicon-steel frame), a modified intramedullary interlocking-nail (i.e., a high permeability silicon-steel sheet is fixed on the nail), and measurement electronics. When the interlocking-nail travels through the air gap of the c-shaped electromagnet, the silicon-steel sheet concentrates the gap’s magnetic flux and thereby the pick-up coil receives more flux. Consequently, due to the electromagnetic induction, the coil produces a larger voltage output. By analyzing the voltage output, the silicon-steel sheet with the screw hole is located and targeted. According to these, in this paper, we modify the targeting method and apply it to develop a passive, powerless, and wireless sensing element with a new, cheap, easy, and compatible electromagnetic targeting system which can combine the EBUS radial probe for real-time navigating/targeting diagnosis for peripheral lung tumors/lesions.

## 2. Design

### 2.1. Targeted Object

Standard/Clinical EBUS radical probe’s guide sheath (type: k-203, Olympus Co., Tokyo, Japan) are used for the navigation/targeting. Because the EBUS radial probe, forceps, and guide sheath are well-aligned before navigating/targeting, the real-time location of the guide sheath is an important reference for navigating and thus is selected as the target for the targeting system. Based on the above information and specifications, the targeting principle and corresponding system are appropriately designed (described follows).

### 2.2. Targeting Principle

**Figure 1:**
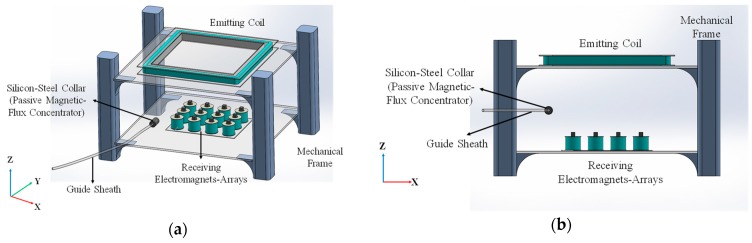
The illustration of the passive magnetic-flux concentrator based electromagnetic targeting system for endobronchoscope: (**a**) isometric view and (**b**) cross-sectional view of the system.

The system, shown in Figure 1, consists of a magnetic-flux emitting coil, a magnetic-flux receiving electromagnets-array, and a passive magnetic-flux concentrator (i.e., a high permeability silicon-steel collar) embedded in a guide sheath. For system testing, an official 3D model of bronchial tree which is used to train medical doctors and operators in hospitals is used to test the system.

**Figure 2:**
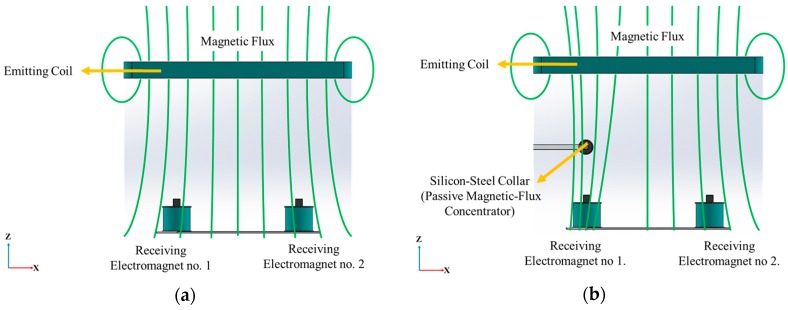
The targeting principle: (**a**) magnetic flux is produced in the space between the emitting coil and the receiving electromagnets-array. (**b**) When the passive magnetic-flux concentrator (silicon-steel collar) travels to the space/location above electromagnet number 1, the magnetic flux is concentrated by the collar while the other magnetic flux above other electromagnets are not concentrated by the collar.

The targeting principle of the system is shown in Figure 2. As shown in Figure 2a, an AC current is applied to the emitting coil to produce a corresponding AC magnetic flux. Consequently, the magnetic flux is received by the receiving electromagnets-array. Due to the electromagnetic-induction, each receiving electromagnet produces a corresponding voltage output. As shown in Figure 2b, when the guide sheath with the silicon-steel collar is inserted into the 3D model of bronchial tree and travels through the space above the receiving electromagnet number 1, the silicon-steel collar concentrates the magnetic flux and thereby the magnetic flux received by the receiving electromagnet number 1 is increased. Due to this, the magnitude of the voltage output in receiving electromagnet number 1 is increased. However, at this moment, the magnetic flux in the space above receiving electromagnet number 2 is not concentrated (because there is no collar there). Thus, the voltage outputs in the other receiving electromagnets are unchanged. By analyzing the voltage changes in receiving electromagnets, the location of the silicon-steel collar is targeted, and thus the location of the guide sheath is also targeted. Successfully targeting/locating guide sheath means successfully targeting/locating the EBUS radial probe in the 3D model of a bronchial tree.

### 2.3. System Architecture

#### 2.3.1. Design of Signal Processing by Using Labview

**Figure 3:**
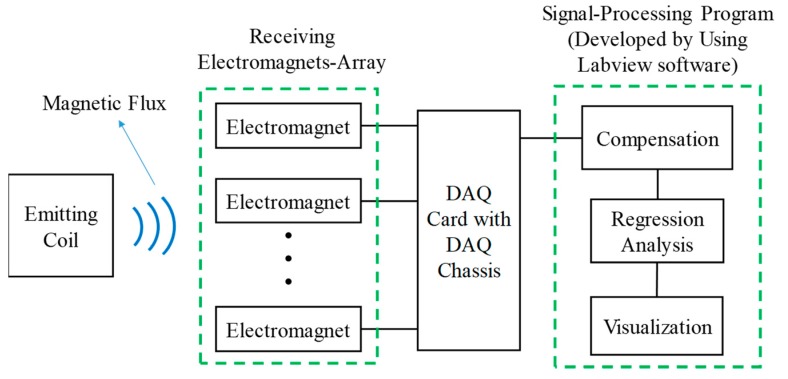
The architecture of the system.

**Figure 4:**
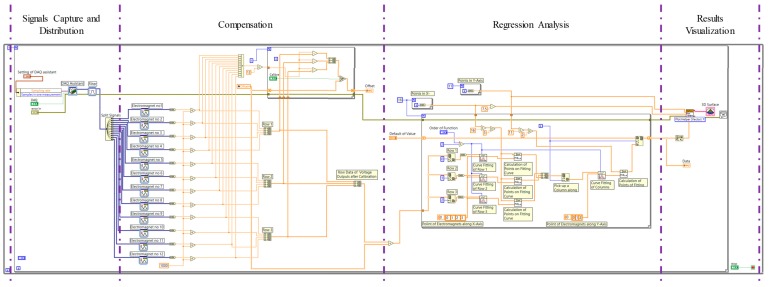
The block diagram of signal-processing program developed by using Labview software.

System architecture is illustrated as Figure 3. The block diagram of a signal-processing program is shown in Figure 4. The emitting coil generates magnetic flux. Consequently, the receiving electromagnets detect the flux and thus produce voltage outputs by the electromagnetic induction. To analyze the voltage output of the receiving electromagnet, a data acquisition card (DAQ card) with a data acquisition chassis (DAQ chassis) and a signal-processing program (developed by using Labview 2018 software, National Instruments, Austin, Tex., USA) are used. Due to practical fabrication issue of electromagnets, voltage output induced by each receiving electromagnet (subjected to the same magnetic flux) may be slightly different (i.e., slightly different output under same input). To eliminate the slight difference, we used the DAQ card to measure the voltage output of receiving electromagnets before the collar is inserted into the bronchi tree (i.e., before detecting the collar). These voltage outputs are defined as “background voltage outputs”. The DAQ card sequentially collects the background voltage outputs, converts them into digital data, and imports the digital data to a signal-processing program. The signal-processing program averages peak-to-peak values of the background voltage outputs and subsequently calculates offset value. Finally, the values of background voltage outputs are added/subtracted by the offset value to achieve the above difference elimination. Therefore, before detecting the collar, the values of voltage outputs of each electromagnet are the average value (without difference). Consequently, without differences, the system can start to detect the collar. When collar travels between the coil and the receiving electromagnets, the voltage outputs of the electromagnets are changed. These changed voltage outputs are defined as “detection voltage–outputs”. Subsequently, the DAQ card measures detection voltage–outputs, converts them into digital data, and finally transfers the data to the signal-processing program. The signal-processing program conducts a regression analysis to correlate detection voltage–outputs and collar’s location information, and consequently plots the correlated results for visualization purpose in clinical diagnosis. Even though the Labview software provides visual block diagrams for us to rapidly develop the signal-processing program, the detailed calculation of regression is not shown with the block diagrams in Figure 4. Therefore, we have to provide the detailed calculation of the regression analysis in the next paragraph for general validation purposes.

#### 2.3.2. Calculation Method of Regression Analysis

Before the regression analysis, we define directions along row and column of the electromagnets-array as *x*-axial and *y*-axial directions, respectively. Furthermore, according to the *x*–*y*-directions, the voltage outputs produced by the electromagnets-array are arranged as a data array which has the same *x*–*y*-layout as the electromagnets-array. When the rearranged data array is imported to regression analysis, the data along the *x*-axial direction (i.e., the data in rows) are sequentially selected to perform two-order polynomial fitting function to obtain *x*-axial fitting curves. The function is described below:

(1) $$f=a_{0}+a_{1}x+a_{2}x^{2},$$

where f is voltage output, *a*_0_, *a*_1_, and *a*_2_ are coefficients of polynomial function, and *x* is the *x*-axial coordinate point of the receiving electromagnet.

To obtain the above coefficients, we use the least squares method. Thus, the polynomial function is transformed to the below equation:

(2) $$S\left(a_{0},a_{1},a_{2}\right)=\sum_{i}{\left[f_{i}-\left(a_{0}+a_{1}x_{i}+a_{2}x_{i}{}^{2}\right)\right]}^{2},$$

where S is the sum of squared difference between the values of imported voltage outputs and predicted voltage outputs by polynomial function, *i* is the number of electromagnets along the *x*-axial direction, and *x_i_* is the *x*-axial coordinate point of the electromagnet number *i*. By the least squares method, when sum of squared difference is minimum, the *a*_0_, *a*_1_, and *a*_2_ are coefficients of the best fitting curve. Due to this, the derivatives of S function (for *a*_0_, *a*_1_, and *a*_2_) are set as zero to minimize the sum of squared difference, as shown below in Equation (3). Thus, the corresponding solutions of the equations are the coefficients of the best fitting curve:

(3) $$\{\begin{array}{c} \frac{\partial S\left(a_{0},a_{1},a_{3}\right)}{\partial a_{0}}=0\\ \frac{\partial S\left(a_{0},a_{1},a_{3}\right)}{\partial a_{2}}=0\\ \frac{\partial S\left(a_{0},a_{1},a_{3}\right)}{\partial a_{3}}=0 \end{array}.$$

The coefficients obtained from the Equation (3) are substituted back into Equation (1) to obtain the fitting curves along the *x*-axial direction. Through the fitting curves, we can predict more completed voltage outputs along the *x*-axial direction. The predicted voltage outputs are subsequently rearranged into another array. The data along the *y*-axial (i.e., data in column) in this rearranged array is selected and applied to the same procedure above of the regression analysis to obtain the fitting curves along the *y*-axial direction. Finally, the results of regression analysis are exported and plotted for further visualization. Note: for the detailed procedure of the above regression analysis, please see the supplementary material.

## 3. Fabrication

Fabrication process of the system is divided into five parts: (I) a passive magnetic-flux concentrator (i.e., a high permeability silicon-steel collar) fixed onto a guide sheath, (II) an emitting coil, (III) a receiving electromagnets-array, (IV) measurement circuits/electronics, and (V) an aluminum-poly(methyl 2-methylpropenoate) (PMMA) mechanical frame.

**Figure 5:**
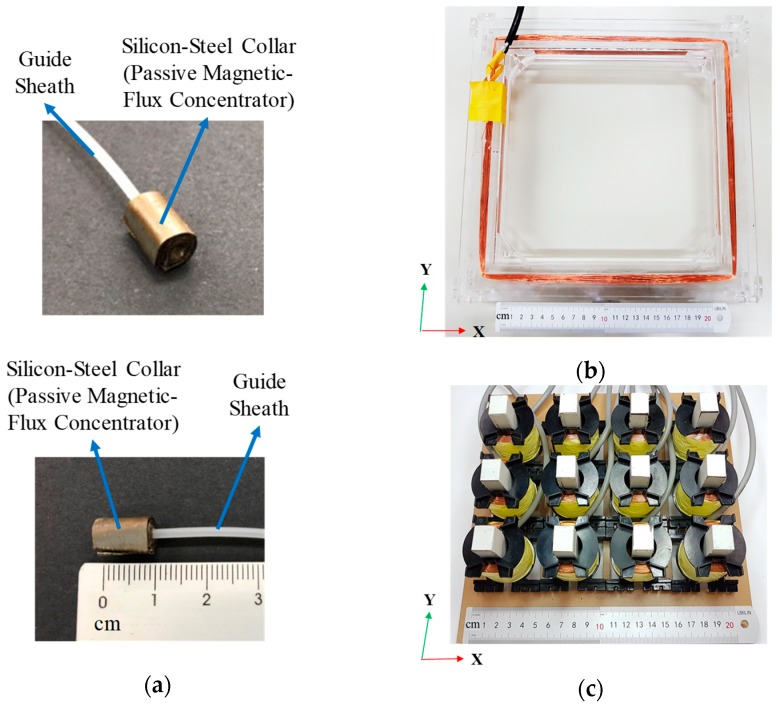
The fabrication results of major components of the system: (**a**) the passive magnetic-flux concentrator (i.e., the high permeability silicon-steel collar) fixed onto a guide sheath, (**b**) the emitting coil, (**c**) the receiving electromagnets-array.

For the first part, silicon-steel sheets (type: 35Z155, Yung–Chin Silicon Steel Co., Ltd., New Taipei City, Taiwan) were altered by using mechanical machining. After this, we stacked four silicon-steel sheets (thickness of each sheet is 0.35 mm) as a collar and fixed it on the end of a commercial smallest guide sheath (type: k-203, Olympus Co., Tokyo, Japan) corresponding to the smallest probe (i.e., EBUS radial probe, Olympus Co., Tokyo, Japan). The diameter and length of the silicon-steel collar is 6 mm and 10 mm, respectively, as shown in Figure 5a.

For the second part, the fabrication of the magnetic-flux emitting coil is described below. We used the milling machine to fabricate the PMMA square frame (length and width: 27 cm and 27 cm). AWG25 enameled wire is wound on the PMMA square frame as the coil (turns of the coil is 300 turns), as shown in Figure 5b.

For the third part, the receiving electromagnets were fabricated by using coils and magnetic cores. The coils (Type: GC-PQ5050-1000TS, G-Chen Enterprise co. Ltd., Hisnchu, Taiwan) were fabricated by AWG25 enameled wires. The turn of each coil is 1000. The outer diameter, inner diameter, and height of each coil is 3.8 cm, 2.1 cm, and 3.6 cm, respectively. After this, 26 pieces of high permeable silicon-steel sheets (model: 35Z155, Yung–Chin Silicon Steel Co., Ltd., New Taipei City, Taiwan) were bound as a stack. The length, width, and thickness of each stack is 4 cm, 1 cm, and 1.1 cm, respectively. One stack (used as a magnetic core) was co-axially placed inside one coil to form one electromagnet. In total, 12 receiving electromagnets were fabricated and assembled on a PMMA board as the receiving array. The center-to-center and edge-to-edge distances between each electromagnet in the array are 5 cm and 1.7 cm, respectively. The fabrication results of the receiving electromagnets-arrays are shown in Figure 5c.

Regarding the fourth part (measurement electronics/circuits), a function generator, a data acquisition card (type: NI-9206, National Instruments, Austin, TX, USA) with data acquisition chassis (model: NI-cDAQ 9174, National Instruments, Austin, TX, USA), and a computer were used for the measurement. The function generator was connected to the emitting coil to produce an AC magnetic field. The data acquisition card (DAQ card) was used to measure the induced voltage of each receiving electromagnet and subsequently store the induced voltage data in the computer for analysis.

**Figure 6:**
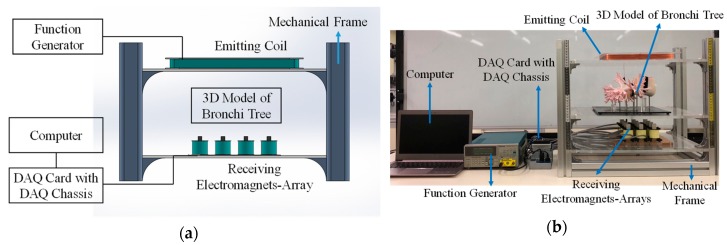
The targeting system after assembling: (**a**) the illustration and (**b**) the photograph of the system.

Finally, aluminum extrusion beams and PMMA plates were used to fabricate the mechanical frame. The mechanical frame was used to assemble the above parts as the complete tracking system shown in Figure 6. The distance between the emitting coil and the receiving electromagnets-array is 25 cm.

## 4. Testing Procedure

**Figure 7:**
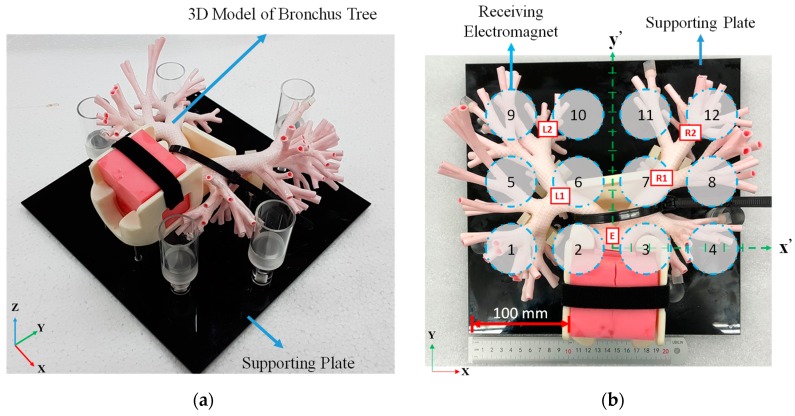
Official/clinical 3D model of bronchus tree for training medical doctors and operators in hospitals: (**a**) isometric view and (**b**) top view showing the relative location between each receiving electromagnet (beneath the model) and each critical entry/intersection location for collar-targeting test (inside the model). *note: the *X*–*Y*–*Z* coordinate system in the figure is used as a general reference indicating overall direction/orientation. The *x*′–*y*′–*z*′ coordinate system in the figure is used to specifically indicate the *x*-, *y*-, and *z*- axial directions for targeting purpose in the bronchial-tree model. (Note: for enlarged Figure 7b, please see the supplementary material).

Before medical doctors and operators are qualified to perform an official/clinical EBUS diagnosis for potential lung tumors/lesions in a patient, the 3D model of bronchus tree is used to train the medical doctors and operators. If our system is able to perform targeting in the 3D model, this indicates that our system is able to assist medical doctors and operators to perform targeting in vivo experiments or in clinical protocols. Therefore, we test our system (i.e., navigating/targeting the guide sheath) by using an official 3D model of bronchus tree as an ex-vivo experiment (type: LM-099, Koken co., Ltd., Tokyo, Japan; Figure 7, a standard model used in hospitals to train medical doctors and operators).

According to the procedure of the diagnosis, the EBUS radial probe is inserted from the mouth, through trachea, and eventually to the bronchi. The intersections in bronchi are important locations for navigating/targeting the probe. Thus, the carina of trachea is defined as the main entry point (i.e., named as location E). The intersections of left primary bronchi and left secondary bronchi, left tertiary bronchi and left bronchioles, right secondary bronchi and right tertiary bronchi, and right tertiary bronchi and right bronchioles are defined as Location L1, L2, R1, and R2, respectively. Blue dash circled regions in the bronchi-model plane (shown in Figure 7b) are projection of the receiving electromagnets beneath the bronchi model. The receiving electromagnets-array is set beneath the bronchi model by aligning the center between electromagnet no. 2 and no. 3 to the center of the trachea. The aligned co-center is projected on the supporting plate of the model as original point (0, 0, 0) of the relative coordinate system x′–y′–z′, as shown in Figure 7b. According to the setup and the relative coordinate system x′–y′–z′, the location E, L1, L2, R1, R2 is at point (−5, 5, 90), (−45, 40, 75), (−55, 100, 65), (35, 55, 85), and (60, 90, 60), respectively (Note: the unit of value for above locations is mm; In addition, please see supplementary material for why these points are quantized by 5 mm). After this, the system is ready for testing.

**Figure 8:**
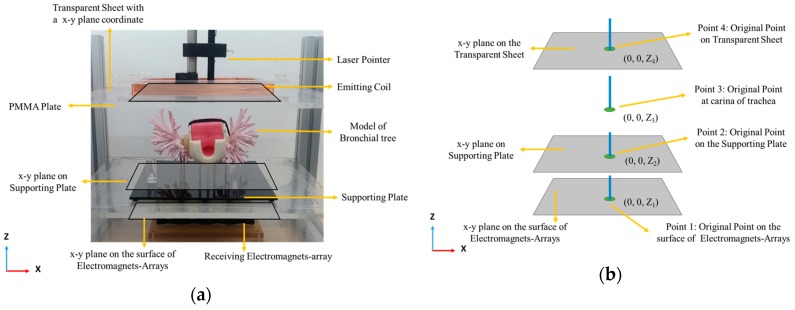

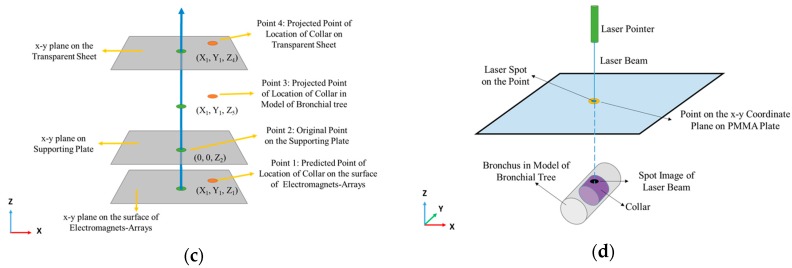
The photograph and illustration of the three parallel *x*–*y* planes and their coordinates for our targeting system: (**a**) the photograph shows each *x*–*y* plane on the (PMMA) plate, supporting plate, and surface of the electromagnets-array, (**b**) the illustration shows original point in each *x*–*y* plane and at carina of trachea, (**c**) the illustration shows predict point and projected point of location of collar on each plane, and (**d**) the illustration of the “projection” that is the method to determine a *z*-coordinate by using a laser pointer.

For testing, as shown in Figure 6, we use a function generator to apply an AC current with frequency 500 Hz and amplitude of 30 mA to the emitting coil to emit magnetic flux. While emitting magnetic flux, the concentrator travels through location E, L1, L2, R1 and R2 in sequence. Simultaneously, the voltage outputs of the receiving electromagnets-array are recorded by using a DAQ card and consequently conducted by Labview 2018 software (National Instruments, Austin, Tex., USA). The voltage outputs are continuously recorded 10 times when the collar is at each location. When the collar is physically targeted by the system, the targeted point on the fitting curve of voltage outputs is virtually projected to the top of the bronchus-tree model as the predicted coordinate point. The detailed method to determine the *x*-, *y*-, and *z*- coordinate points are described below. First, we fix a transparent sheet with an *x*–*y* plane coordinate on a PMMA plate that is fixed to the mechanical frame of the targeting system. This forms three parallel *x*–*y* planes respectively on the transparent sheet, the supporting plate of bronchial model, and the surface of electromagnets-array, as shown in Figure 8a. After this, we align the carina of trachea of bronchial tree model and the center between electromagnet no. 2 and no. 3 in the electromagnets-array to the original points of their plane coordinates. Thus, coordinates of the model, supporting plate of the model, and the targeting system have the same *x*–*y*–*z* orientations and similar original points (i.e., the same *x* and *y*, but different *z*), shown in Figure 8b. Next, when the collar travels in the bronchial tree model, the targeting system can predict the coordinate points of the collar, shown as point 1 in Figure 8c. Point 1 is correlated to the transparent sheet to obtain the corresponding predicted coordinate point in the plane coordinate on the transparent sheet (i.e., point 4 in Figure 8c). After this, we use a laser pointer to vertically shoot a laser spot though these coordinate points (as shown in Figure 8d) to the bronchial tree model (defined as “projection” method). When a laser pointer vertically shoots a laser beam to a point on the plane, if the collar is vertically beneath the shot point in the model, the spot-image of the laser beam will be aligned on the collar, as shown in Figure 8d. Due to this, the spot-image also has the same *x*–*y* coordinate points as the shot point. Thus, the *x*–*y* coordinates point of the collar is obtained (i.e., the 2D (*x*, *y*) coordinates of the collar in the 3D model is obtained). After projection, the location of the laser spot on the bronchial tree model is defined as point 3 in Figure 8c. Finally, we measure the *z*-axial-height between the location on the model and the supporting plate (i.e., Z_5_ subtracts Z_2_). After this, we set the original point of whole system to the original point on the *x*–*y* plane of the supporting plate (i.e., point 2 in Figure 8c). Based on this setting, the value of the *z*-coordinate of the predicted location of the collar will be the *z*-axial-height. That is, the predicted locations in 3D model are obtained. Due to this, the targeting error is calculated through the vector below in Equation (4):

(4) $$\vec{e_{D}}=\vec{P_{e}}-\vec{P_{a}},$$

where $\vec{e_{D}}$ is the targeting error, $\vec{P_{e}}$ is the predicted coordinate point by using our system, and $\vec{P_{a}}$ is the actual coordinate point of the collar (these are all in vector form).

## 5. Result and Discussion

**Figure 9:**
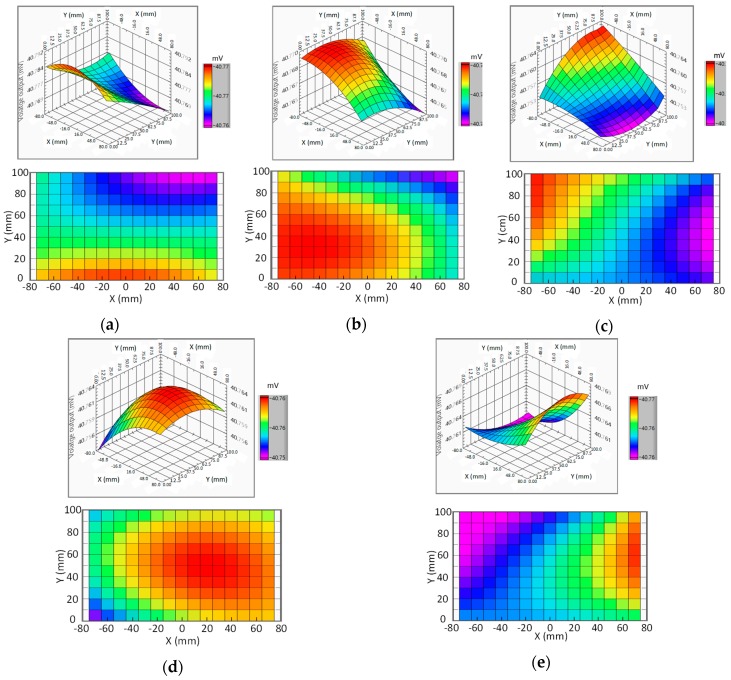
The test results of the system: the isometric view and top-plane view of curve-fitting-predicted voltage outputs of the receiving electromagnets when the collar moves to (**a**) location E, (**b**) location L1, (**c**) location L2, (**d**) location R1, and (**e**) location R2 in the bronchi-tree model.

Figure 9 shows test results of the system (note: for the raw data of these results, please see the supplementary material). Table 1 is the summary of actual coordinate points, estimated/predicted coordinates points, and targeting errors. According to the results, when the silicon-steel collar is at location E (entry point; close to carina of trachea), most of the magnetic flux is concentrated by the collar and thus the electromagnet no. 2 (which is located nearest to the location E) receives more magnetic flux than other electromagnets. Consequently, by electromagnetic induction, the electromagnet no. 2 produces the largest voltage output than the other electromagnets. Furthermore, after signal processing, the predicted results are plotted. Based on the results, predicted voltage output at point (−5, 0) is locally larger than the other points, as shown in Figure 9a. The location of the point showing the local largest voltage output in the plot is subsequently projected to the bronchi tree model to estimate/predict the actual collar coordinate point in the bronchi-tree model. This estimated/predicted coordinate point in the model is approximately at (−5, 0, 90). The actual coordinate of the collar at location E is at point (−5, 5, 90). After comparing, the estimated/predicted coordinate point and the actual coordinate points are very close. This shows that our approach can successfully target the collar at location E. After testing at the location E, we move the collar into the left lung for different location tests.

When the collar continuously travels to location L1 (intersection of left primary bronchi and left secondary bronchi), the magnetic flux is concentrated by electromagnet no. 6, and thus the voltage output of electromagnet no. 6 is the largest among all electromagnets. After analyzing the fitting curve, the predicted voltage output at point (−45, 30) is the largest. At this moment, the electromagnet no. 2 receives less magnetic flux and thus produces lower voltage output (i.e., the voltage output at previously predicted point (−5, 0) decreases when the collar moves away). These results show that the predicted location of the collar is moved from previous coordinate point (−5, 0) to current coordinate point (−45, 30), as shown in Figure 9b. After analysis, the estimated/predicted coordinate point in 3D model is at (−45, 30, 75). In fact, the actual coordinate of the collar at location L1 is at point (−45, 40, 75) in the 3D model. After comparing, because the estimated/predicted coordinate point is close to the actual coordinate point, we can conclude that the collar is successfully targeted at region L1.

Similarly, when the collar continuously travels to location L2 (i.e., intersection between left tertiary bronchi and left bronchioles), the point showing largest voltage output is between electromagnet no. 9 and no. 10. After analyzing the fitting curve, the coordinate point (−75, 100) has the largest predicted voltage output, as shown in Figure 9c. After analysis, the estimated/predicted coordinate point in 3D model is at (−75, 100, 70). In fact, the actual coordinate point of the collar at location L2 is at (−55, 100, 65). After comparing, because the estimated/predicted coordinate point is roughly close to the actual coordinate point, we can conclude that the collar could be targeted at region L2.

For the contrast test, we remove the collar from the left lung, and subsequently insert the collar into the right lung and repeat the above procedure. When the collar moves to location R1 (i.e., intersection between right secondary bronchi and right tertiary bronchi), the electromagnet no. 7 produces the largest voltage output. After curve fitting, the predicted coordinate point of the collar is at (25, 50), as shown in Figure 9d. After analysis, the estimated/predicted coordinate point in 3D model is at (25, 50, 80). In fact, the actual coordinate point of the collar at location R1 is at (35, 55, 85). After comparing, the estimated/predicted coordinate point and the actual coordinate point are close. This indicates that the collar is successfully targeted at region R1.

Similarly, when the collar continuously moves from location R1 to location R2 (i.e., intersection of right tertiary bronchi and right bronchioles), the electromagnet no. 12 produces the largest voltage output. After curve fitting, the estimated/predicted coordinate point of the collar is at (75, 60), as shown in Figure 9e. After analysis, the estimated/predicted coordinate point in 3D model is at (75, 60, 45). In fact, the actual coordinate point of the collar at location R2 is at (60, 90, 60). After comparing, the estimated/predict coordinate point is roughly close to the actual coordinate point. This means that the collar could be targeted at region R2. 

In addition, when the collar is targeted at each location, we calculate the targeting error at each location. Finally, for each location, the predicted coordinate point, the actual coordinate point, and corresponding targeting errors are summarized in Table 1. According to these results, the average targeting errors on the *x*-, *y*-, and *z*-axes are 9, 10, and 5 mm, respectively. These results show that we are able to successfully target the collar (sheath) by analyzing the predicted voltage outputs of all points in the curve–fitting plot. This also means that our proposed design/approach is successfully validated through the tests.

Moreover, according to the method to determine the *x*-, *y*-, and *z*-coordinate points, the predicted *z*-axis value is not obtained by the voltage output of the electromagnets. Thus, the *z*-value highly depends on the shape of the bronchial tree model. It is meaningless to discuss the *z*-value for our system. In fact, the *x*- and *y*-values are more important than *z*-values because a lung is typically featured with an *x*–*y* planar geometric-shape. Due to this, when the bronchoscope travels in the bronchial tree, the bronchoscope always encounters a carina that divides one bronchus to two bronchi (i.e., left and right bronchi). Therefore, to target the bronchoscope, it is important to distinguish the *x*- and *y*-coordinates rather than the *z*-coordinate of the bronchoscope. However, in research perspectives, the *z*-coordinate values still can be used as an assisting reference to assist the targeting of the bronchoscope for special cases of lung geometry in clinics. According to this reason, we still show the *z*-value of error in this paper to provide assisting information for the targeting of a bronchoscope. 

**Figure 10:**
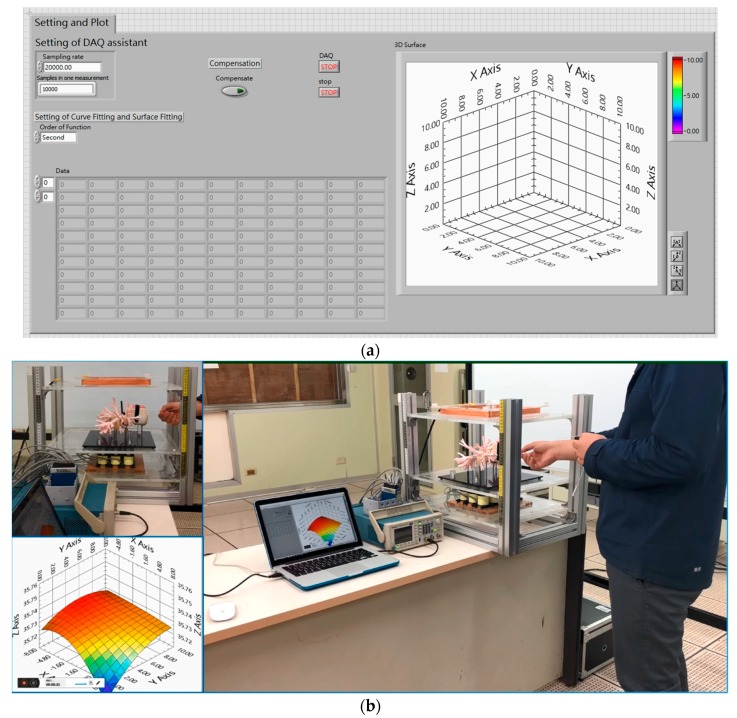
Demonstration of using the user–interface program in a targeting test: (**a**) program overview, (**b**) video-demonstration of using the program in a real-time targeting test (note: the program is developed by using the Labview program).

Furthermore, based on above signal-processing procedure and corresponding curving–fitting results, we develop a user–interface program for medical doctors and operators to ease the targeting approach in clinical diagnosis, as shown in Figure 10. That is, when these users use our system to target the collar traveling in the bronchi-tree model, voltage outputs of receiving electromagnets are immediately/automatically curve-fitted/plotted on the program’s interface to show the largest voltage output to indicate the collar’s real-time location. Figure 10a shows the user–interface program of the system. Figure 10b is a screenshot of a video-demonstration of using the program in the targeting test. For the demonstration video, please see the supplementary material section.

We also notice that some researchers developed a theoretical model for their electromagnetic targeting/navigation approaches [7,8]. In their approaches, the targeted object is an inductive inductor-capacitor (LC) resonant coil (note: When the LC coil is subjected to a magnetic field, an inductive resonant magnetic field is induced in the LC coil due to the electromagnetic induction. That is, without any power supplied to the LC coil, the LC coil can produce the inductive resonant magnetic field in itself). A detailed sensing principle of their approaches is described below. When an emitting coil produces a pulse magnetic field, the LC coil receives this pulse magnetic field. Consequently, an inductive magnetic field at the LC coil’s resonant frequency is induced in the LC coil. When inducing the resonant magnetic field, the LC coil behaves like an “active” magnetic diploe which can actively emit flux. The magnitude/strength of the magnetic dipole is measured by their system and subsequently is analyzed by the same system to obtain the location information of the LC coil through the theoretical model. However, in our system, the targeted object is a silicon-steel collar (i.e., passive magnetic flux concentrator) rather than the LC coil. Because the silicon collar under the magnetic field will not induce an inductive resonant magnetic field in collar itself, the collar can only be regarded as a “passive” magnetic dipole that cannot actively emit flux. Due to this discrepancy, their model cannot be applied to our case. Thus, we have to develop a new theoretical model for our approach and modify a corresponding signal-processing program of our system. However, developing our own theoretical model needs a long-tern effort and would be another full length article. Due to this, herein this paper, we use a simple signal-processing program with a curve-fitting approach to analyze the location of our targeted object. Except the modeling method, we also provide several methods to improve the accuracy of our system. The first method is increasing diameter of receiving electromagnets. When the diameter increases, the voltage output also increases. According to this relation, if a small change of magnetic flux induced by the small movement of the collar, the electromagnet with a larger diameter is able to convert the small change of the magnetic flux into a large change of voltage output. Due to this, the small movement of the collar is able to be detected by a system and thus the accuracy can be increased. The second method is increasing the number of electromagnets within the same planar area of the electromagnets-array (i.e., increasing the sensing-resolution). When the amount of receiving electromagnets is increased in the same area, the pitches between each electromagnet on the *x*-axis and *y*-axis are decreased (i.e., the resolution of sensing are increased). Due to this, the system can record more experimental results along the *x*-axis and *y*-axis in the same area and consequently use more experimental results to conduct the more accurate regression analysis. Thus, the accuracy of system increases. However, we have to consider the trade-off issue between the diameter and the number of electromagnets. In addition to the two parameters above, it is better to investigate the other parameters influencing the targeting accuracy together at the same time (i.e., considering not only the receiving electromagnets, but also emitting electromagnets, etc.) and consequently optimize these parameters from system perspectives. The third method is improving the regression analysis. We can use other functions, such as Gauss distribution or higher order polynomial function, to conduct the regression analysis. Due to this, the regression analysis may match the experimental results more accurately. Thus, local-largest-point predicted by regression analysis may approach more closely to the actual local-largest-point, even when the actual local-largest-point still cannot be calculated and plotted. Due to this, the accuracy is increased. For the detailed discussion for above methods, please see supplementary material.

After we carefully review our approach and results, we think the error of L2 may cause insufficient targeting. This error is due to the location of L2 which is on the edge of the working space of our targeting system. This causes that the targeting accuracy is not very accurate. Nevertheless, we think the current accuracy of our system is still acceptable. The reason is described below. To evaluate whether our system has an acceptable accuracy for the requirement for hospital use, we provide a summary of accuracy of commercial electromagnetic tracking systems for hospitals (in terms of errors) and subsequently compare our system with these commercial systems. Nowadays, there are two commercial tracking systems: “SPiN Thoracic Navigation System” provided by Veran Medical Technologies Co. and “superDimension” provided by Medtronic Co. Both electromagnetic tracking systems have been evaluated by clinical tests and used in hospitals for official diagnosis. This indicates that both tracking systems satisfy the requirement of hospital use. To highlight the accuracy, the errors of our and their electromagnetic tracking systems are summarized in Table 2. The error of the SPiN Thoracic Navigation System is 2.6 mm. The error of superDimension is 6.1 mm. However, even though they provide their error for their targeting system, the calculation methods of errors are not reported. Moreover, the manufacturers of these systems would develop their own mathematical or theoretical analysis in the signal-processing program to eliminate errors of the targeting system. However, their analysis methods for these systems are also not available. Due to these, when comparing the commercial electromagnetic tracking systems to our system, all we can do is to list all of our errors in each axis for comparison. In Table 2, we notice that the errors of our system on the *x*- and *y*-axes are about 9 and 10 mm, respectively. It seems that the accuracy may not be sufficient for targeting. Nevertheless, when medical doctors use the targeting system in diagnosis, the medical doctors can always refer to the CT scan image of the bronchi tree anytime during the diagnosis [25,27]. By overlapping tumor’s/lesions’ locations in the CT scan image and targeted locations obtained by targeting system, the medical doctors are able to realize the actual moving path of the bronchoscope in the patient’s lung. Moreover, to avoid any hurt caused from the bronchoscope to the bronchi tree during diagnosis, the bronchoscope can only move slowly and is constrained by the bronchial wall. When the error significantly influences the targeting or the predicted location of the bronchoscope is outside of the bronchial tree, medical doctors can withdraw the bronchoscope from this location back to previous location according to the record of traveled path (above-mentioned). When the bronchoscope is back at a previous location (sort of resetting), the medical doctors can re-guide the bronchoscope again and re-predict the location of the bronchoscope though the moving path of the collar (re-navigation; like using Google map for re-navigation/re-calculating the route while driving a car). In addition, during targeting in real clinical operations in hospitals, the travel-path of the collar and the CT scan image of the bronchial tree can be referenced together by the medical doctors to judge whether the travel-path of the collar is reasonable or needs an adjustment (like using a Google map while driving a car, drivers can always judge whether they should directly follow the proposed routes or adjust by referring to a real map with surrounding observation). Furthermore, when considering a typical distribution of bronchial tree in a general human lung, one bronchus is always subdivided into two smaller bronchi (for example, the left primary bronchus is subdivided into two secondary bronchi). The subdividing wall is called a carina. Furthermore, the distribution of the two smaller bronchi will always be that one bronchus is on the left-hand side while another bronchus is on the right-hand side. Thus, when medical doctors guide the bronchoscope moving in bronchial tree for targeting, the bronchoscope will encounter a carina and thus the medical doctors must decide to move the bronchoscope to the left-hand-side or right-hand-side bronchi. If the targeting system does not predict the location of the bronchoscope very accurately, the medical doctors could know the location of the bronchoscope in the bronchus by referring to the CT scan image of lungs anytime to recall the travelled path of the bronchoscope and consequently adjust the moving direction of the bronchoscope. Due to this reference and consequential adjustment, medical doctors can guide (and simultaneously adjust) the bronchoscope to move along the correct path to the bronchi with lung-cancer tumors. Due to these, even though the targeting system does not predict the location of bronchoscope very accurately, the bronchoscope still can be guided to the location of lung-cancer tumors in the bronchial tree. According to these, we think our system has an acceptable accuracy for the requirement for hospital use. 

Finally, we evaluate the bronchus that allows our collar to travel by surveying some lecture for diameters of different bronchi. Researchers reported that diameters of a primary bronchi, secondary bronchi, tertiary bronchi, and bronchioles are about 12.2, 8.3, 5.6, and 4.5 mm, respectively [37]. We summarize the diameter of different bronchi in Table 3. According to their study, the collar in this paper only can reach the secondary bronchi. From these results, if we need to access further bronchi by our system, the diameter of collar must be reduced. The first method is using different materials owning better sensing response to make smaller collars. The second method is to optimize the receiving electromagnets to get a better sensitivity by using theoretical analysis [38]. For the detailed of these two methods, please see supplementary material. 

## 6. Conclusions

We successfully demonstrated a novel electromagnetic targeting system using a magnetic-flux concentrator to target an endobronchoscope used in lung cancer tumor/lesion diagnosis. The system was tested by using the official/clinical bronchial-tree model which is used to train medical doctors and operators in hospitals. According to the test results, the targeting errors on the *x*-, *y*-, and *z*-axes are 9 mm, 10 mm, and 5 mm, respectively. Due to this, the system successfully targeted the EBUS radial probe’s guide sheath in the model (through locating the silicon-steel collar fixed on the guide sheath). Targeting the guide sheath means that the consequently diagnosis tools (such as EBUS radial probe and biopsy forceps) can be simultaneously targeted. In the future, we will continuously improve the system and apply the system to animal tests (and eventually to the human body in clinical cases).

## Figures and Tables

**Table 1:** Summary of testing results: Actual coordinate points, predicted coordinate points, and targeting errors.

	Actual Coordinate Point of Collar (Unit: mm)	Predicted Coordinate Point of Collar (Unit: mm)	Targeting Error (Unit: mm)	Average Targeting-Error (Unit: mm)
Location E	(−5, 5, 90)	(−5, 0, 90)	*x*-axial: 0 *y*-axial: 5 *z*-axial: 0	*x*-axial: 9 *y*-axial: 10 *z*-axial: 5
Location L1	(−45, 40, 75)	(−45, 30, 75)	*x*-axial: 0 *y*-axial: 10 *z*-axial: 0	
Location L2	(−55, 100, 65)	(−75, 100, 70)	*x*-axial: 20 *y*-axial: 0 *z*-axial: 5	
Location R1	(35, 55, 85)	(25, 50, 80)	*x*-axial: 10 *y*-axial: 5 *z*-axial: 5	
Location R2	(60, 90, 60)	(75, 60, 45)	*x*-axial: 15 *y*-axial: 30 *z*-axial: 15	

**Table 2:** Comparison of errors of commercial and our electromagnetic targeting systems.

	SPiN Thoracic Navigation System (Veran Medical Technologies) [36]	Super Dimension (Medtronic) [36]	Our Work
Error	2.6 mm	6.1 mm	*x*-Axial: 9 mm *y*-Axial: 10 mm *z*-Axial: 5 mm

**Table 3:** Typical diameter of bronchus [37].

Type of Bronchus	Outer Diameter of Bronchus
Primary Bronchi	12.2 mm
Secondary Bronchi	8.3 mm
Tertiary Bronchi	5.6 mm
Bronchioles	4.5 mm

## Supplementary Materials

The following are available online at https://www.mdpi.com/1424-8220/19/23/5105/s1, Figure S1: The enlarged figure of official/clinical 3D model of bronchus tree for training medical doctors and operators in hospitals, Figure S2: The photograph of the plane coordinate on the transparent sheet, Figure S3: The illustration of the method of curve fitting, Figure S4: The results of curve fitting: the isometric view and top-plane view of curve-fitting-predicted voltage outputs of the receiving electromagnets, Figure S5: The result of predicted voltage output at different location with calibration (upper figure) and without calibration (lower figure), Figure S6: The block diagrams of the curve fitting, Figure S7: The measured voltage output and offset values of receiving electromagnets when the collar is at different locations, Figure S8: The result of the curve fitting from x-axis to y-axis when the collar at location 1, Figure S9: The result of the curve fitting from y-axis to x-axis when the collar at location 1, Figure S10, The result of the curve fitting from x-axis to y-axis when the collar at location 2, Figure S11: The result of the curve fitting from y-axis to x-axis when the collar at location 2, Figure S12: The result of the curve fitting from x-axis to y-axis when the collar at location 3, Figure S13: The result of the curve fitting from y-axis to x-axis when the collar at location 3, Figure S14: The result of the curve fitting from x-axis to y-axis when the collar at location 4, Figure S15: The result of the curve fitting from y-axis to x-axis when the collar at location 4, Figure S16: The result of the curve fitting from x-axis to y-axis when the collar at location 5, Figure S17: The result of the curve fitting from y-axis to x-axis when the collar at location 5, Figure S18: The raw data of the location of E, Figure S19: The raw data of the location of L1, Figure S20: The raw data of the location of L2, Figure S21: The raw data of the location of R1, Figure S22: The raw data of the location of R2, Figure S23: The test setup for sensing performance by using different materials for the collar, Table S1: Typical diameter of bronchus, Table S2: The summary of the change of voltage output of different materials, Video S1: Demonstration Video.
