# Supplementary material for: Passive Magnetic-Flux-Concentrator Based Electromagnetic Targeting System for Endobronchoscopy
Source: Sensors (Basel). 2019 Nov 21;19(23):5105. doi: 10.3390/s19235105 (PMC6928937; doi:10.3390/s19235105)
Supplement: Supplementary file 1 [file sensors-19-05105-s001.zip › sensors-560828-supplementary/sensors-560828-supplementary-proof done.pdf]

sensors

MDPI

Supplementary Material

Passive Magnetic-Flux-Concentrator Based Electromagnetic Targeting System for Endobronchoscopy

Chin-Chung Chen [1,#], Ching-Kai Lin [1,2,3,4,#], Chen-Wei Chang [1], Yun-Chien Cheng [1], Jia-En Chen [1], Sung-Lin Tsai [1] and Tien-Kan Chung [1,5,*]

1 Department of Mechanical Engineering, National Chiao Tung University, Hsinchu 30010, Taiwan; chinchung.me02g@nctu.edu.tw (C.-C.C.); vanhalen@ntuh.gov.tw (C.-K.L.); wychang.me05g@g2.nctu.edu.tw (C.-W.C.); yccheng@nctu.edu.tw (Y.-C.C.); kh14760.me06g@nctu.edu.tw (J.-E.C); peter.me03@g2.nctu.edu.tw (S.-L.T)
2 Department of Internal Medicine, National Taiwan University Hospital, Taipei 10002 Taiwan
3 Department of Internal Medicine, National Taiwan University Hospital Hsin-Chu Branch, Hsinchu 30059, Taiwan
4 Department of Medicine, National Taiwan University Cancer Center, Taipei 10672, Taiwan
5 International College of Semiconductor Technology, National Chiao Tung University, Hsinchu 30010, Taiwan
* Correspondence: tkchung@nctu.edu.tw; Tel.:886-3-5712121 #55116
\# Co-First Author (Equal Contribution)

1. Enlarged Figure of the Figure 7b in Our Paper

Due to the limitation of paper's margin, the value of ruler in Figure 7b in this paper is invisible. Thus, in following (see below Figure S1), we provide the enlarged Figure 7b to show values on the ruler.

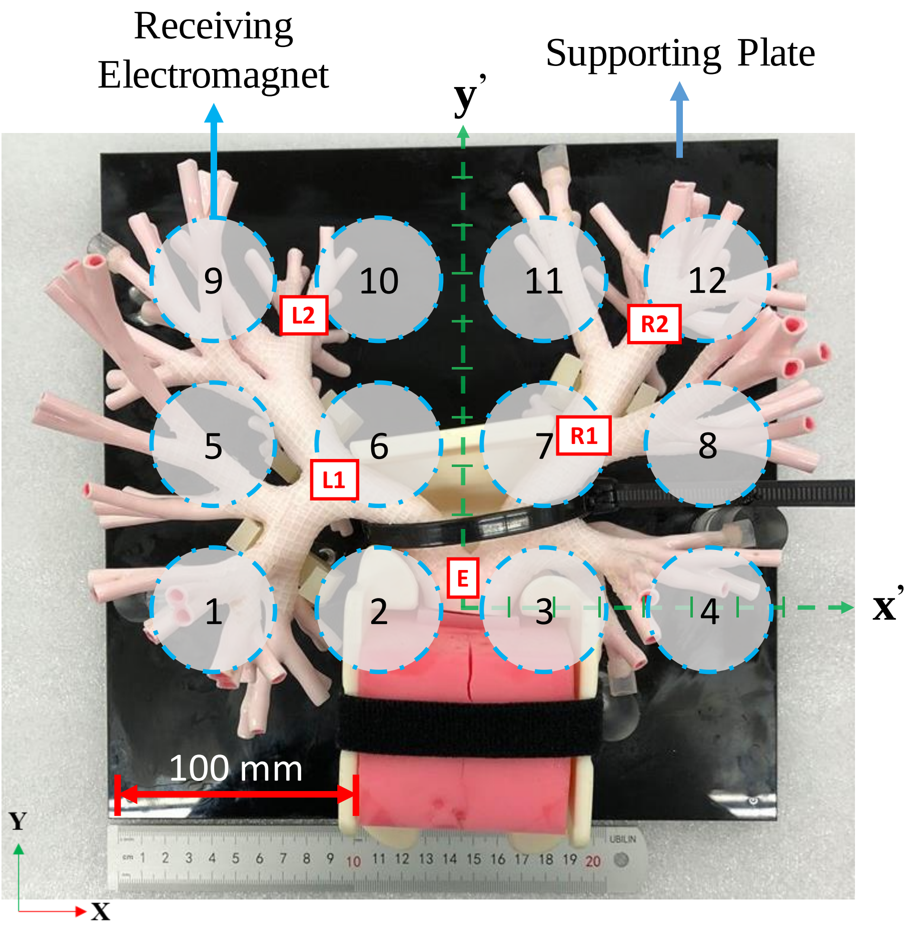

Figure S1. The enlarged figure of official/clinical 3D model of bronchus tree for training medical doctors and operators in hospitals: top view showing the relative location between each receiving electromagnet (beneath the model) and each critical entry/intersection location for collar-targeting test (inside the model).

2. Detailed Explanations for: Why Every Physical Position Value is Quantized by 5 and How the Accuracy of Physical Position Measurement is. Why Predicted Position Value is Quantized by 10.

The reason for quantized by 5 is due to the measurement method of physical position. As the method in original paper, both coordinate points of predicted location and actual location are determined by using laser-beam measurement. That is, we use the laser pointer to shoot the laser beam through the plane coordinate on the transparent sheet to the actual location. The point on the plane coordinate on the transparent sheet (where the laser beam penetrates though) is the coordinate points of the actual location. In this measurement, because the length of minimum grid in the plane coordinate on the transparent sheet is 5 mm (see below Figure S2), the minimum scale of the plane coordinate is 5 mm accordingly. Thus, every physical position value is quantized by 5 mm. Due to this minimum scale, the accuracy of physical position measurement is also 5 mm.

Regarding the predicted position value, according to the testing procedure in original paper, the direction along length of the electromagnets-array is x-axial direction (see Figure 5c in paper). When we conduct the regression analysis along x-axis, we use 16 points for calculation. Furthermore, the length of electromagnets-array (Note: length between the center of electromagnet no. 1 and electromagnet no. 4) is 150 mm. Due to this, the interval of any of two-neighbor-points along x-axis is 10 mm. Consequently, for example, if the original point is on the electromagnet no. 1, x coordinate points in sequential will be 0, 10, 20, …, 150 mm, respectively. In fact, in our paper, we set the original point on the center between electromagnet no 2 and no 3. Due to the shift of the original point (which

is 75 mm), the x coordinate points in sequential are subtracted by 75. Therefore, the x coordinate points in sequential become -75, −65, … 0, …, 65, 75 mm, respectively. According to this reason, it seems that the estimated values are quantized by 5. However, in fact, the increment of the predicted values is 10 mm.

(a) (b)

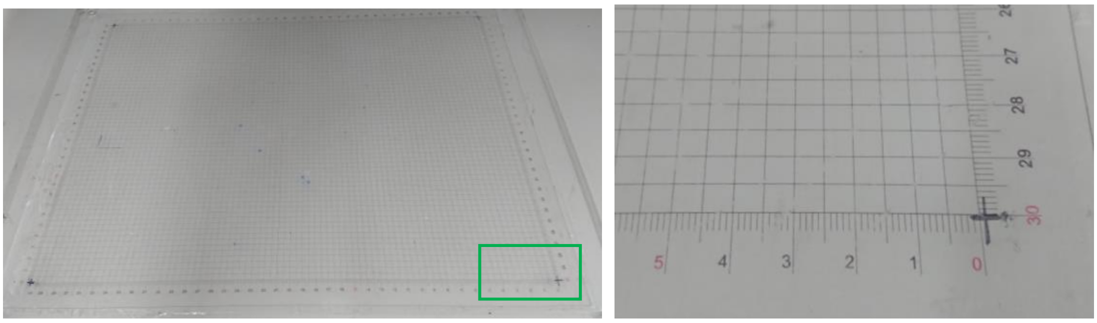

Figure S2. The photograph of the plane coordinate on the transparent sheet: (**a**) side view and (**b**) enlarged view of the green section in (a).

3. The Detailed Method of Regression Analysis and Comparison between Regression Analysis for x-Axis First and y-Axis first.

In following, we provide detailed procedure to explain how we conduct the curve fitting of the regression analysis. Before the curve fitting, due to practical fabrication issue of the electromagnets, voltage output induced by each receiving electromagnet (which is subjected to the same magnetic flux) may be slightly different (i.e., slightly different output under same input). To eliminate the slight difference, we measure the voltage output of receiving electromagnets before the testing. These voltage outputs are defined as "background voltage outputs", and consequently used to calculates offset value for each electromagnet. After the offset values are obtained, the system is used to target the collar. During the targeting, voltage outputs of electromagnets-array are obtained and subsequently arranged into a data array based on the layout of electromagnets-array, as shown in Figure S3a. In Figure S3a, the V_x is the measured voltage output of electromagnet no. x. However, as we mentioned, the slight difference of the electromagnets needs to be eliminated. Thus, the measured voltage output is subtracted by the offset values. After subtraction, we arrange the voltage outputs into another array, as shown in Figure S3b. In Figure S3b, the $V_{offset\text{-}x}$ is the offset value of the electromagnet no. x. Next, we pick up subtracted voltage outputs in each row (i.e., along x-axial direction of the system). For example, the row (V_1-$V_{offset\ 1}$, V_2-$V_{offset\ 2}$, V_3-$V_{offset\ 3}$, V_4-$V_{offset\ 3}$) are picked, as shown in the green circle in Figure S3b. After this, we conduct the curve fitting for the picked voltage outputs in each row, respectively. In the curve fitting, because the interval of each point is 1 cm and the length of the electromagnets-array is 150 mm, we import total 16 points (i.e., 0 cm, 10 mm, 20 mm, 30 mm, …., 150 mm) to estimate the voltage outputs. After the estimation, predicted voltage outputs in each row is obtained (for example, row ($V_{X(1,\ 1)}$, $V_{X(1,\ 2)}$ … $V_{X(1,\ 15)}$, $V_{X(1,\ 16)}$). Consequently, we rearrange these predicted voltage outputs into another array, as shown in figure S3c. In Figure S3c, the $V_{x(i,j)}$ is the predicted voltage output at row i and column j obtained by curve fitting for x-axis. After this, we pick up predicted voltage output in each column (along y-axial direction of the system from $V_{x(i,\ j)}$ array), for example, column ($V_{X(1,\ 1)}$, $V_{X(2,\ 1)}$, $V_{X(3,\ 1)}$) as shown in the green circle in Figure S3c. After picking up the column, we use the values in each column to conduct the curve fitting for y-axis, respectively. After the fitting curve for y-axis is obtained, similarly, because the interval of each point is 1 cm and the width of the electromagnets-array is 100 mm, we import 11 points (i.e., points at 0 mm, 1 mm, 2 mm, 3 mm, …., 100 mm) into the fitting curve for y-axis to calculate the predicted voltage output for each column. After the estimation, the predicted voltage outputs in each column are obtained and shown in Figure S3d (for example, column ($V_{Y(1,\ 1)}$, $V_{Y(2,\ 1)}$ … $V_{Y(10,\ 1)}$, $V_{Y(11,\ 1)}$). In Figure S3d, the $V_{Y(i,\ j)}$ is the predicted voltage output of at row i and column j obtained by curve

fitting for y-axis. Finally, the data in $V_{Y(i,\ j)}$ array and the corresponding x-y coordinate points are plotted as the results shown in below Figure S4 and the Figure 9 in original paper.

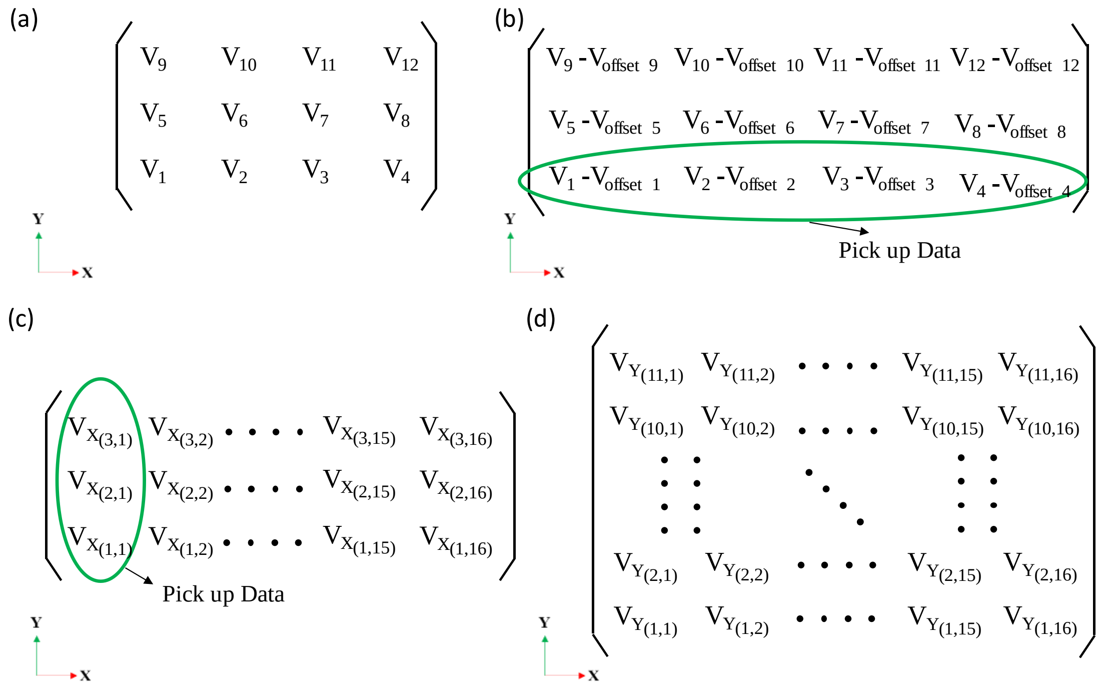

Figure S3. The illustration of the method of curve fitting: (**a**) The measured voltage outputs of the electromagnets-array are arranged as a data-array. (**b**) The measured voltage outputs subtract the offset values; which are used to conduct the curve fitting for x-axis. (**c**) The predicted voltage outputs which are obtained from the fitting curve for x-axis are used to conduct curve fitting for y-axis. (**d**) The predicted voltage output obtained from the fitting curve of y-axis. Please see corresponding paragraphs for the detailed description of these figures (note: the orientation of the x-y coordinate in each figure and the electromagnets-array are the same).

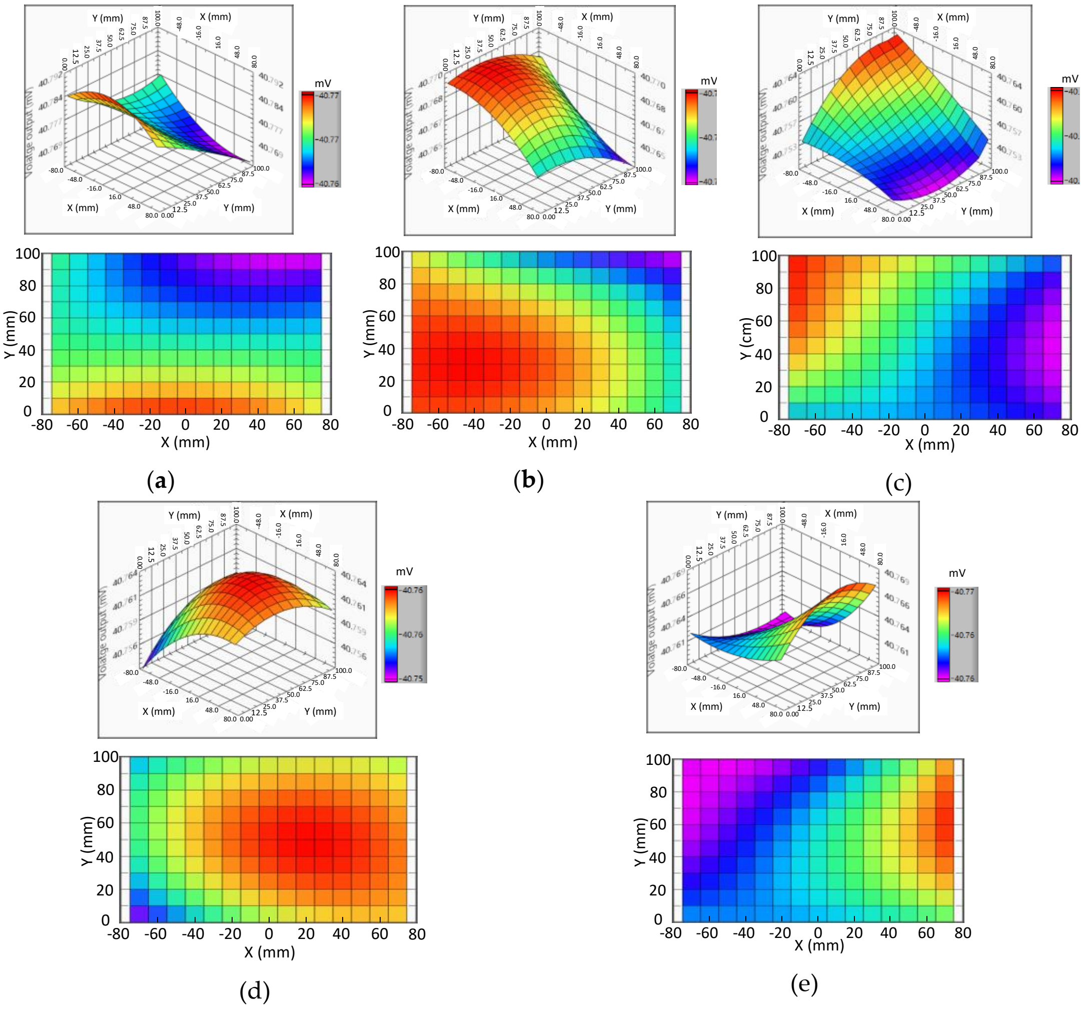

Figure S4. The results of curve fitting: the isometric view and top-plane view of curve-fitting-predicted voltage outputs of the receiving electromagnets when the collar moves to (**a**) location E, (**b**) location L1, (**c**) location L2, (**d**) location R1, and (**e**) location R2 in the bronchi-tree model.

Regarding the performing curve fitting for y-axis first (and x-axis later), we redo another experiment because the offset values used to calibrate the system were conducted in the signal process program and cannot be exported as a text file in previous experiments in our paper. Thus, the offset values are not available. Due to this, in the redo experiment, we export the offset values as a text file during the experiment. [As a note, below paragraph addresses the issue of offset values for calibration: If the results of predicted voltages are not calibrated by offset values, the results of curve fitting would not be able to indicate the location of collar by local maximum voltage output. Figure S5 shows the results of curve fitting with and without calibration at location E, L1, L2, R1, and R2. When the collar is at location E, the result of curve fitting with calibration has only one local maximum voltage output, as shown in upper figure of Figure S5a. Thus, the collar is able to be targeted. However, without calibration, the result has two local maximum voltage outputs, as shown in lower figure of Figure S5a. We are not able to determine which local maximum voltage output is induced by the collar. According to this result, the location of collar is not able to be targeted by the result without calibration. Furthermore, even though the collar is at different location, the results of curve fitting without calibration are almost the same. Due to this, in our signal-processing program, we need the offset values to conduct the curve fitting, otherwise the slight difference between each electromagnet will influence the result of curve fitting]. Due to these, we redo the experiment to record both of raw data (measured voltage outputs) and offset values to conduct two regression

analyses (i.e., curve fitting from x-axis to y-axis and from y-axis to x-axis). In the re-do experiment, we test out system when the collar is at 5 locations and conduct two regression analyses [the coordinate points of 5 locations 1, 2, 3, 4, and 5 are (0, 5), (−45, 30), (−60, 65), (35, 55) and (55, 85), respectively. To conduct two curve fittings, we modify and develop the signal-processing program (developed by using Labview 2019). The block diagrams of the two curve fittings in Labview software are shown in Figure S6].

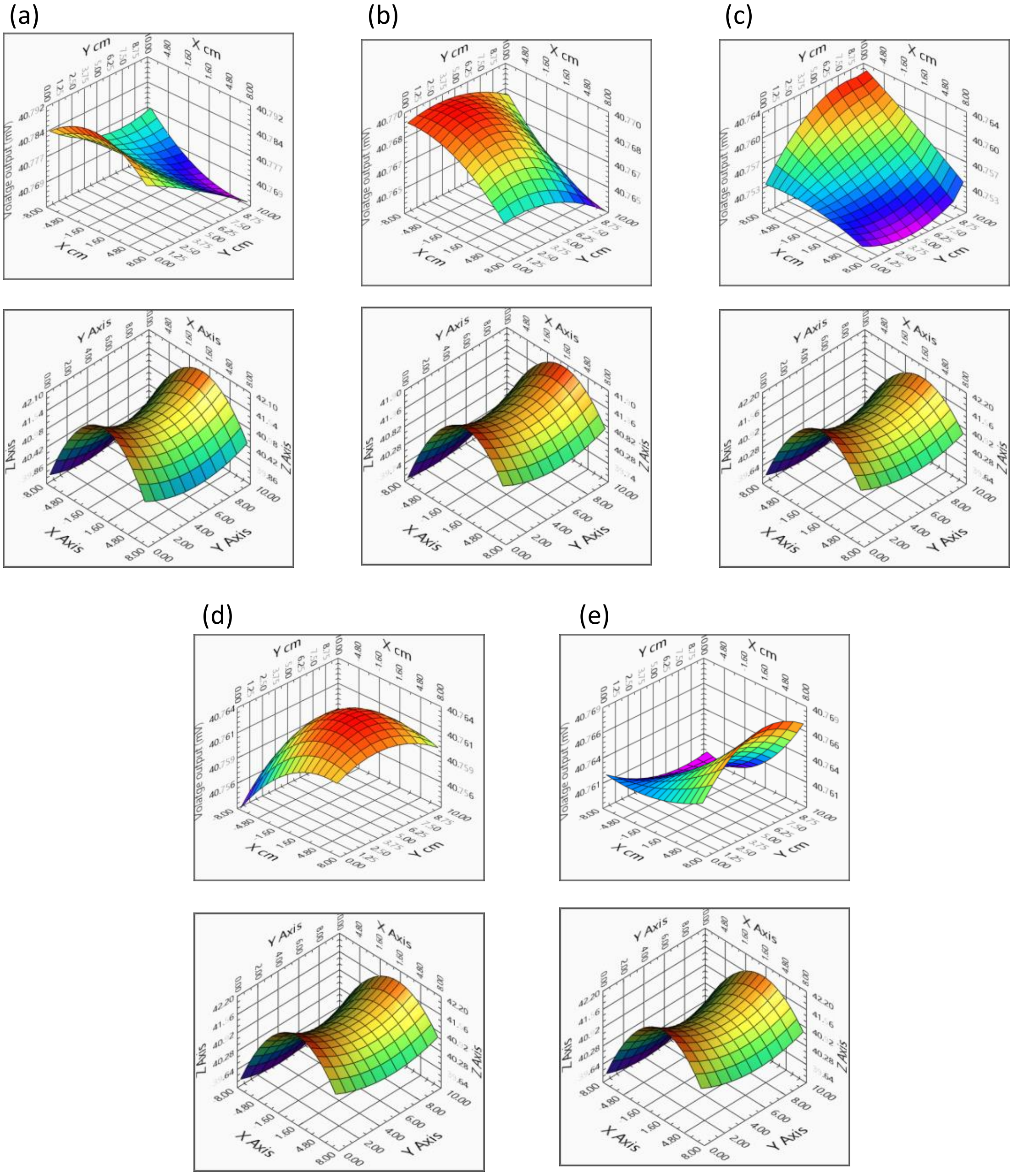

Figure S5. The result of predicted voltage output at different location with calibration (upper figure) and without calibration (lower figure): (**a**) location E, (**b**) location L1, (**c**) location L2, (**d**) location R1, and (**e**) location R2.

(a)

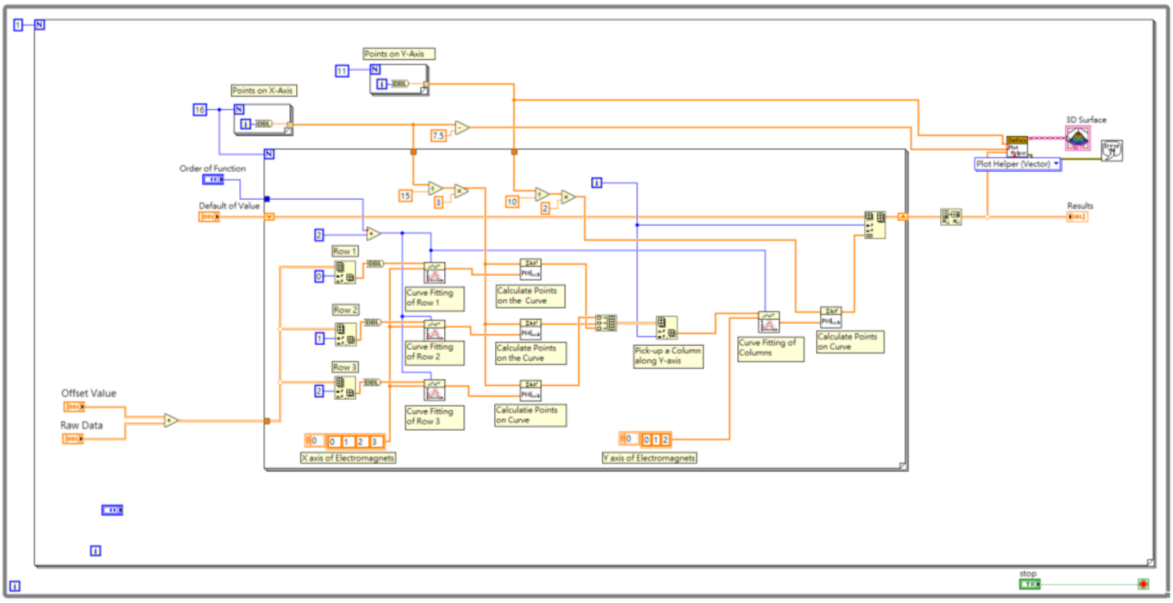

(b)

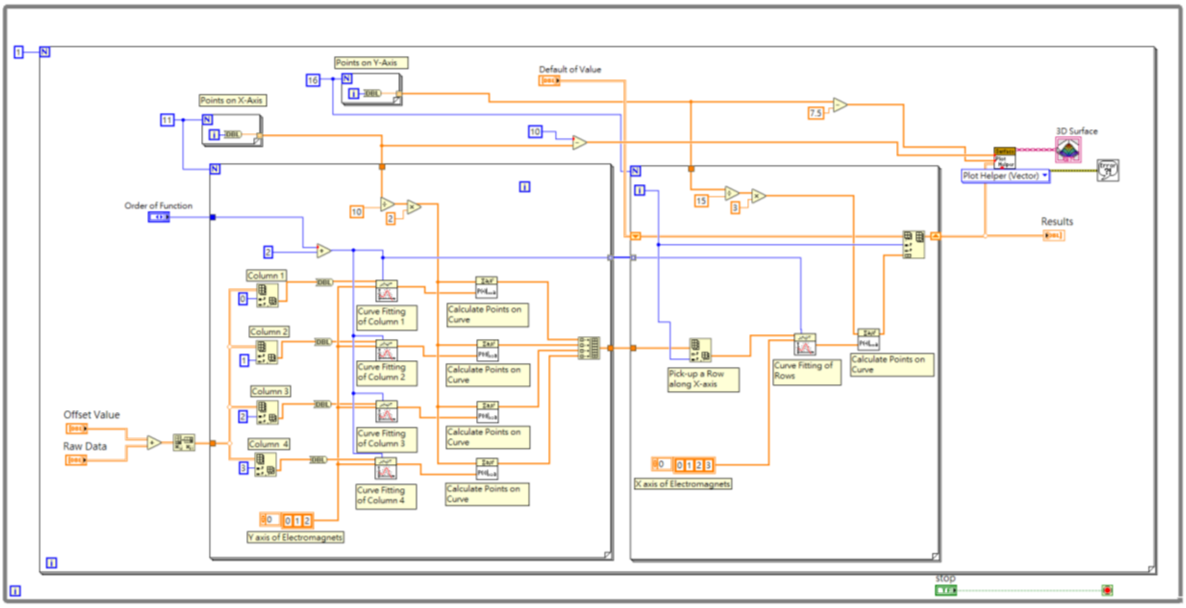

Figure S6. The block diagrams of the curve fitting which starts: (**a**) from x-axis to y-axis and (**b**) from y-axis to x-axis.

After we modify the signal-processing program, we test the system and analyze the output signals by using above two curve-fitting programs. Figure S7 shows the raw data (measured voltage outputs) and offset values at each location. After we obtain the raw data and the offset value, we import them to the signal-processing program to conduct the curve fitting. The results of two curve fittings are shown from Figure S8 to Figure S17. In these figures, the values which are highlighted by red color are the maximum predicted voltage output. According to results, when the collar is at location 1, 2, 3, 4 and 5, the predicted location obtained by curve fitting from x-axis to y-axis are (20, 0), (−75, 30), (−75, 80), (75, 65) and (75, 100), respectively. The predicted location obtained by curve fitting from y-axis to x-axis is (20, 0), (−75, 30), (−75, 80), (75, 65) and (75, 100), respectively (Note: If the largest estimated values of voltage outputs on two neighboring points are the same, we choose the central point between the two points as the point having local largest estimated value). According

to the results, the predicted locations of the collar are the same when using curve-fitting starting from x-axis to y-axis or from y-axis to x-axis. Due to this, consequently, the predicted voltage outputs are also the same. That is, the predicated locations and predicted voltage outputs are the same when using curve-fitting starting from x-axis to y-axis or from y-axis to x-axis. According to the results, the averaged x-axis and y-axis targeting errors of curve fitting starting from x-axis to y-axis are 25 mm and 9 mm, respectively. The averaged x-axis and y-axis targeting errors of curve fitting starting from y-axis to x-axis are also 25 mm and 9 mm, respectively. Furthermore, as we mentioned, our system has sufficient accuracy to navigate the bronchoscope even though the averaged targeting error is up to 25 mm. Because the results obtained by both curve fittings (starting from x-axis to y-axis and from y-axis to x-axis) are the same, both curve fitting are applicable and thus we finally choose the original curve fitting (i.e., the one starting from x-axis to y-axis) for our manuscript. Note: we also add the results of the curve fitting starting from y-axis to x-axis as contrast references in the supplementary material. In the future, according to above results, we will continuously improve our signal processing method to increase accuracy for patients' benefits.

(a) Location 1

Measured Voltage output

Y-Axis (mm)				
100	53.2854	57.1865	56.9069	53.9314
50	54.0283	56.6285	56.5122	53.1925
0	53.0909	57.6942	56.4893	52.3045
X-Axis (mm)	75	-25	25	75

Offset Value

1.8282	-2.07698	-1.77041	1.18385
1.07123	-1.52641	-1.41403	1.90291
2.00469	-2.58974	-1.39532	2.79202

(b) Location 2

Measured Voltage output

Y-Axis (mm)				
100	53.2904	57.1892	56.8386	53.7998
50	54.026	56.6556	56.5332	53.1995
0	53.0949	57.5312	56.513	52.307
X-Axis (mm)	75	-25	25	75

Offset Value

1.79606	-2.10505	-1.76153	1.28051
1.06171	-1.56884	-1.45177	1.8775
1.98602	-2.44995	-1.43268	2.76802

(c) Location 3

Measured Voltage output

Y-Axis (mm)				
100	53.2899	57.1847	56.8509	53.8166
50	54.034	56.6525	56.5269	53.199
0	53.1078	57.5624	56.5109	52.3088
X-Axis (mm)	75	-25	25	75

Offset Value

1.79972	-2.10038	-1.76713	1.26124
1.0634	-1.56117	-1.44374	1.88323
1.99097	-2.47435	-1.4256	2.7738

(d) Location 4

Measured Voltage output

Y-Axis (mm)				
100	53.2795	57.1768	56.8625	53.8348
50	54.0247	56.647	56.5381	53.2125
0	53.1021	57.5807	56.5229	52.3227
X-Axis (mm)	75	-25	25	75

Offset Value

1.80335	-2.08994	-1.76918	1.25192
1.06424	-1.55474	-1.43847	1.88589
1.98688	-2.48797	-1.42479	2.77282

(e) Location 5

Measured Voltage output

Y-Axis (mm)				
100	53.285	57.1757	56.8883	53.8726
50	54.0261	56.6391	56.5293	53.2121
0	53.1002	57.6264	56.5214	52.3327
X-Axis (mm)	75	-25	25	75

Offset Value

1.81107	-2.08094	-1.79345	1.22697
1.069	-1.54272	-1.42524	1.89717
1.99607	-2.53056	-1.40994	2.78355

Layout of Receiving Electromagnets

No. 9	No. 10	No. 11	No. 12
No. 5	No. 6	No. 7	No. 8
No. 1	No. 2	No. 3	No. 4

Figure S7. The measured voltage output and offset values of receiving electromagnets when the collar is at different locations: (**a**) location 1, (**b**) location 2, (**c**) location 3, (**d**) location 4 and (**e**) location 5.

Unit: mV

Y-Axis (mm)

100	55.0972	55.0979	55.0985	55.099	55.0994	55.0996	55.0997	55.0997	55.0995	55.0992	55.0988	55.0983	55.0976	55.0969	55.0959	55.0949
90	55.0972	55.0976	55.098	55.0982	55.0983	55.0983	55.0983	55.0981	55.0978	55.0974	55.0969	55.0964	55.0957	55.0949	55.094	55.093
80	55.0974	55.0977	55.0979	55.0979	55.0979	55.0978	55.0977	55.0974	55.097	55.0966	55.0961	55.0954	55.0947	55.0939	55.093	55.0921
70	55.098	55.0982	55.0983	55.0983	55.0983	55.0981	55.0979	55.0976	55.0972	55.0967	55.0961	55.0955	55.0948	55.094	55.0931	55.0921
60	55.0988	55.0991	55.0992	55.0993	55.0993	55.0991	55.0989	55.0987	55.0983	55.0978	55.0972	55.0966	55.0958	55.095	55.0941	55.0931
50	55.0999	55.1003	55.1006	55.1009	55.101	55.1009	55.1008	55.1006	55.1003	55.0999	55.0993	55.0987	55.0979	55.0971	55.0961	55.095
40	55.1013	55.102	55.1026	55.103	55.1033	55.1035	55.1035	55.1035	55.1032	55.1029	55.1024	55.1018	55.101	55.1001	55.0991	55.0979
30	55.103	55.1041	55.105	55.1058	55.1064	55.1068	55.1071	55.1072	55.1071	55.1069	55.1065	55.1059	55.1051	55.1042	55.1031	55.1018
20	55.1049	55.1065	55.108	55.1092	55.1102	55.1109	55.1115	55.1118	55.1119	55.1118	55.1115	55.111	55.1102	55.1092	55.1081	55.1067
10	55.1071	55.1094	55.1114	55.1131	55.1146	55.1158	55.1167	55.1173	55.1177	55.1178	55.1176	55.1171	55.1163	55.1153	55.114	55.1125
0	55.1096	55.1127	55.1154	55.1177	55.1197	55.1214	55.1227	55.1237	55.1244	55.1246	55.1246	55.1242	55.1235	55.1224	55.121	55.1192
	-75	-65	-55	-45	-35	-25	-15	-5	5	15	25	35	45	55	65	75

X-Axis (mm)

Figure S8. The result of the curve fitting from x-axis to y-axis when the collar at location 1. From the results, the predicted location is at (20, 0). (Note: due to the values at (15, 0) and (25,0) are the same, we chose the center of these two points as the predicted location).

Unit: mV

Y-Axis (mm)

100	55.0972	55.0979	55.0985	55.099	55.0994	55.0996	55.0997	55.0997	55.0995	55.0992	55.0988	55.0983	55.0976	55.0969	55.0959	55.0949
90	55.0972	55.0976	55.098	55.0982	55.0983	55.0983	55.0983	55.0981	55.0978	55.0974	55.0969	55.0964	55.0957	55.0949	55.094	55.093
80	55.0974	55.0977	55.0979	55.0979	55.0979	55.0978	55.0977	55.0974	55.097	55.0966	55.0961	55.0954	55.0947	55.0939	55.093	55.0921
70	55.098	55.0982	55.0983	55.0983	55.0983	55.0981	55.0979	55.0976	55.0972	55.0967	55.0961	55.0955	55.0948	55.094	55.0931	55.0921
60	55.0988	55.0991	55.0992	55.0993	55.0993	55.0991	55.0989	55.0987	55.0983	55.0978	55.0972	55.0966	55.0958	55.095	55.0941	55.0931
50	55.0999	55.1003	55.1006	55.1009	55.101	55.1009	55.1008	55.1006	55.1003	55.0999	55.0993	55.0987	55.0979	55.0971	55.0961	55.095
40	55.1013	55.102	55.1026	55.103	55.1033	55.1035	55.1035	55.1035	55.1032	55.1029	55.1024	55.1018	55.101	55.1001	55.0991	55.0979
30	55.103	55.1041	55.105	55.1058	55.1064	55.1068	55.1071	55.1072	55.1071	55.1069	55.1065	55.1059	55.1051	55.1042	55.1031	55.1018
20	55.1049	55.1065	55.108	55.1092	55.1102	55.1109	55.1115	55.1118	55.1119	55.1118	55.1115	55.111	55.1102	55.1092	55.1081	55.1067
10	55.1071	55.1094	55.1114	55.1131	55.1146	55.1158	55.1167	55.1173	55.1177	55.1178	55.1176	55.1171	55.1163	55.1153	55.114	55.1125
0	55.1096	55.1127	55.1154	55.1177	55.1197	55.1214	55.1227	55.1237	55.1244	55.1246	55.1246	55.1242	55.1235	55.1224	55.121	55.1192
	-75	-65	-55	-45	-35	-25	-15	-5	5	15	25	35	45	55	65	75

X-Axis (mm)

Figure S9. The result of the curve fitting from y-axis to x-axis when the collar at location 1. From the results, the predicted location is at (20, 0). (Note: due to the values at (15, 0) and (25, 0) are the same, we chose the center of these two points as the predicted location).

Unit: mV

Y-Axis (mm)

100	55.0808	55.0812	55.0815	55.0817	55.0818	55.0817	55.0816	55.0813	55.0809	55.0805	55.0799	55.0791	55.0783	55.0774	55.0763	55.0752
90	55.0828	55.0832	55.0833	55.0834	55.0834	55.0832	55.083	55.0826	55.0821	55.0815	55.0807	55.0799	55.0789	55.0779	55.0767	55.0754
80	55.0846	55.0848	55.0849	55.0848	55.0847	55.0844	55.084	55.0836	55.083	55.0823	55.0814	55.0805	55.0795	55.0783	55.077	55.0756
70	55.086	55.0861	55.086	55.0859	55.0856	55.0853	55.0848	55.0842	55.0836	55.0828	55.0819	55.0809	55.0798	55.0786	55.0774	55.076
60	55.0872	55.0871	55.0869	55.0866	55.0862	55.0858	55.0852	55.0846	55.0839	55.0831	55.0822	55.0812	55.0801	55.0789	55.0777	55.0763
50	55.088	55.0877	55.0874	55.087	55.0865	55.086	55.0854	55.0847	55.0839	55.0831	55.0822	55.0813	55.0802	55.0791	55.078	55.0767
40	55.0885	55.088	55.0875	55.087	55.0864	55.0858	55.0852	55.0845	55.0837	55.0829	55.0821	55.0812	55.0803	55.0793	55.0783	55.0772
30	55.0886	55.088	55.0874	55.0867	55.086	55.0853	55.0846	55.0839	55.0832	55.0825	55.0817	55.0809	55.0801	55.0793	55.0785	55.0777
20	55.0885	55.0876	55.0868	55.086	55.0853	55.0845	55.0838	55.0831	55.0824	55.0818	55.0811	55.0805	55.0799	55.0793	55.0788	55.0783
10	55.088	55.0869	55.086	55.085	55.0842	55.0834	55.0826	55.082	55.0814	55.0808	55.0803	55.0799	55.0796	55.0793	55.0791	55.0789
0	55.0872	55.0859	55.0848	55.0837	55.0827	55.0819	55.0812	55.0805	55.08	55.0796	55.0793	55.0792	55.0791	55.0791	55.0793	55.0796
	-75	-65	-55	-45	-35	-25	-15	-5	5	15	25	35	45	55	65	75

X-Axis (mm)

Figure S10. The result of the curve fitting from x-axis to y-axis when the collar at location 2. From the results, the predicted location is at (−75, 30).

Unit: mV

Y-Axis (mm)

100	55.0808	55.0812	55.0815	55.0817	55.0818	55.0817	55.0816	55.0813	55.0809	55.0805	55.0799	55.0791	55.0783	55.0774	55.0763	55.0752
90	55.0828	55.0832	55.0833	55.0834	55.0834	55.0832	55.083	55.0826	55.0821	55.0815	55.0807	55.0799	55.0789	55.0779	55.0767	55.0754
80	55.0846	55.0848	55.0849	55.0848	55.0847	55.0844	55.084	55.0836	55.083	55.0823	55.0814	55.0805	55.0795	55.0783	55.077	55.0756
70	55.086	55.0861	55.086	55.0859	55.0856	55.0853	55.0848	55.0842	55.0836	55.0828	55.0819	55.0809	55.0798	55.0786	55.0774	55.076
60	55.0872	55.0871	55.0869	55.0866	55.0862	55.0858	55.0852	55.0846	55.0839	55.0831	55.0822	55.0812	55.0801	55.0789	55.0777	55.0763
50	55.088	55.0877	55.0874	55.087	55.0865	55.086	55.0854	55.0847	55.0839	55.0831	55.0822	55.0813	55.0802	55.0791	55.078	55.0767
40	55.0885	55.088	55.0875	55.087	55.0864	55.0858	55.0852	55.0845	55.0837	55.0829	55.0821	55.0812	55.0803	55.0793	55.0783	55.0772
30	55.0886	55.088	55.0874	55.0867	55.086	55.0853	55.0846	55.0839	55.0832	55.0825	55.0817	55.0809	55.0801	55.0793	55.0785	55.0777
20	55.0885	55.0876	55.0868	55.086	55.0853	55.0845	55.0838	55.0831	55.0824	55.0818	55.0811	55.0805	55.0799	55.0793	55.0788	55.0783
10	55.088	55.0869	55.086	55.085	55.0842	55.0834	55.0826	55.082	55.0814	55.0808	55.0803	55.0799	55.0796	55.0793	55.0791	55.0789
0	55.0872	55.0859	55.0848	55.0837	55.0827	55.0819	55.0812	55.0805	55.08	55.0796	55.0793	55.0792	55.0791	55.0791	55.0793	55.0796
	-75	-65	-55	-45	-35	-25	-15	-5	5	15	25	35	45	55	65	75

X-Axis (mm)

Figure S11. The result of the curve fitting from y-axis to x-axis when the collar at location 2. From the results, the predicted location is at (−75, 30).

Unit: mV

Y-Axis (mm)																
100	55.0984	55.0962	55.0942	55.0924	55.0907	55.0892	55.0879	55.0867	55.0857	55.0848	55.0841	55.0836	55.0832	55.083	55.0829	55.083
90	55.0989	55.0968	55.0948	55.093	55.0913	55.0898	55.0884	55.0872	55.0861	55.0852	55.0844	55.0838	55.0834	55.0831	55.0829	55.0829
80	55.0992	55.0971	55.0951	55.0933	55.0916	55.0901	55.0887	55.0875	55.0864	55.0854	55.0846	55.084	55.0834	55.0831	55.0828	55.0828
70	55.0991	55.097	55.0951	55.0934	55.0917	55.0902	55.0889	55.0876	55.0865	55.0855	55.0847	55.084	55.0834	55.083	55.0827	55.0825
60	55.0986	55.0967	55.0949	55.0932	55.0916	55.0902	55.0888	55.0876	55.0865	55.0856	55.0847	55.084	55.0833	55.0828	55.0824	55.0822
50	55.0979	55.0961	55.0944	55.0928	55.0913	55.0899	55.0886	55.0875	55.0864	55.0854	55.0846	55.0838	55.0831	55.0826	55.0821	55.0818
40	55.0968	55.0952	55.0936	55.0922	55.0908	55.0895	55.0883	55.0872	55.0861	55.0852	55.0843	55.0835	55.0828	55.0822	55.0817	55.0813
30	55.0954	55.0939	55.0926	55.0913	55.09	55.0889	55.0878	55.0867	55.0857	55.0848	55.084	55.0832	55.0825	55.0818	55.0812	55.0807
20	55.0936	55.0924	55.0913	55.0901	55.0891	55.088	55.0871	55.0861	55.0852	55.0843	55.0835	55.0827	55.082	55.0813	55.0806	55.08
10	55.0915	55.0906	55.0897	55.0888	55.0879	55.087	55.0862	55.0854	55.0845	55.0837	55.0829	55.0822	55.0814	55.0807	55.0799	55.0792
0	55.0891	55.0885	55.0878	55.0872	55.0865	55.0858	55.0851	55.0844	55.0837	55.083	55.0823	55.0815	55.0807	55.0799	55.0792	55.0783
	-75	-65	-55	-45	-35	-25	-15	-5	5	15	25	35	45	55	65	75

X-Axis (mm)

Figure S12. The result of the curve fitting from x-axis to y-axis when the collar at location 3. From the results, the predicted location is at (−75, 80).

Unit: mV

Y-Axis (mm)																
100	55.0984	55.0962	55.0942	55.0924	55.0907	55.0892	55.0879	55.0867	55.0857	55.0848	55.0841	55.0836	55.0832	55.083	55.0829	55.083
90	55.0989	55.0968	55.0948	55.093	55.0913	55.0898	55.0884	55.0872	55.0861	55.0852	55.0844	55.0838	55.0834	55.0831	55.0829	55.0829
80	55.0992	55.0971	55.0951	55.0933	55.0916	55.0901	55.0887	55.0875	55.0864	55.0854	55.0846	55.084	55.0834	55.0831	55.0828	55.0828
70	55.0991	55.097	55.0951	55.0934	55.0917	55.0902	55.0889	55.0876	55.0865	55.0855	55.0847	55.084	55.0834	55.083	55.0827	55.0825
60	55.0986	55.0967	55.0949	55.0932	55.0916	55.0902	55.0888	55.0876	55.0865	55.0856	55.0847	55.084	55.0833	55.0828	55.0824	55.0822
50	55.0979	55.0961	55.0944	55.0928	55.0913	55.0899	55.0886	55.0875	55.0864	55.0854	55.0846	55.0838	55.0831	55.0826	55.0821	55.0818
40	55.0968	55.0952	55.0936	55.0922	55.0908	55.0895	55.0883	55.0872	55.0861	55.0852	55.0843	55.0835	55.0828	55.0822	55.0817	55.0813
30	55.0954	55.0939	55.0926	55.0913	55.09	55.0889	55.0878	55.0867	55.0857	55.0848	55.084	55.0832	55.0825	55.0818	55.0812	55.0807
20	55.0936	55.0924	55.0913	55.0901	55.0891	55.088	55.0871	55.0861	55.0852	55.0843	55.0835	55.0827	55.082	55.0813	55.0806	55.08
10	55.0915	55.0906	55.0897	55.0888	55.0879	55.087	55.0862	55.0854	55.0845	55.0837	55.0829	55.0822	55.0814	55.0807	55.0799	55.0792
0	55.0891	55.0885	55.0878	55.0872	55.0865	55.0858	55.0851	55.0844	55.0837	55.083	55.0823	55.0815	55.0807	55.0799	55.0792	55.0783
	-75	-65	-55	-45	-35	-25	-15	-5	5	15	25	35	45	55	65	75

X-Axis (mm)

Figure S13. The result of the curve fitting from y-axis to x-axis when the collar at location 3. From the results, the predicted location is at (−75, 80).

Unit: mV

Y-Axis (mm)

100	55.0885	55.0899	55.0911	55.0923	55.0933	55.0942	55.0949	55.0956	55.0961	55.0964	55.0967	55.0968	55.0968	55.0966	55.0964	55.096
90	55.0889	55.0903	55.0915	55.0926	55.0937	55.0946	55.0954	55.0961	55.0967	55.0971	55.0975	55.0978	55.0979	55.098	55.0979	55.0978
80	55.0891	55.0905	55.0917	55.0928	55.0938	55.0948	55.0956	55.0963	55.097	55.0976	55.098	55.0984	55.0987	55.0989	55.099	55.099
70	55.0891	55.0904	55.0916	55.0928	55.0938	55.0948	55.0956	55.0964	55.0971	55.0977	55.0982	55.0987	55.099	55.0993	55.0995	55.0996
60	55.0888	55.0901	55.0914	55.0925	55.0936	55.0946	55.0954	55.0962	55.097	55.0976	55.0981	55.0986	55.099	55.0993	55.0995	55.0996
50	55.0883	55.0897	55.0909	55.0921	55.0932	55.0942	55.0951	55.0959	55.0966	55.0972	55.0977	55.0982	55.0985	55.0988	55.0989	55.099
40	55.0875	55.089	55.0903	55.0915	55.0926	55.0936	55.0945	55.0952	55.0959	55.0965	55.097	55.0974	55.0977	55.0978	55.0979	55.0979
30	55.0865	55.088	55.0894	55.0906	55.0918	55.0928	55.0936	55.0944	55.0951	55.0956	55.096	55.0963	55.0964	55.0964	55.0964	55.0962
20	55.0853	55.0869	55.0883	55.0896	55.0908	55.0918	55.0926	55.0933	55.0939	55.0943	55.0946	55.0948	55.0948	55.0946	55.0943	55.0939
10	55.0838	55.0855	55.087	55.0884	55.0896	55.0906	55.0914	55.0921	55.0925	55.0928	55.093	55.0929	55.0927	55.0923	55.0917	55.091
0	55.0821	55.0839	55.0856	55.087	55.0882	55.0892	55.09	55.0905	55.0909	55.0911	55.091	55.0907	55.0902	55.0895	55.0886	55.0875
	-75	-65	-55	-45	-35	-25	-15	-5	5	15	25	35	45	55	65	75

X-Axis (mm)

Figure S14. The result of the curve fitting from x-axis to y-axis when the collar at location 4. From the results, the predicted location is at (75, 65). (Note: due to the values at (75, 60) and (75, 70) are the same, we chose the center of these two points as the predicted location).

Unit: mV

Y-Axis (mm)

100	55.0885	55.0899	55.0911	55.0923	55.0933	55.0942	55.0949	55.0956	55.0961	55.0964	55.0967	55.0968	55.0968	55.0966	55.0964	55.096
90	55.0889	55.0903	55.0915	55.0926	55.0937	55.0946	55.0954	55.0961	55.0967	55.0971	55.0975	55.0978	55.0979	55.098	55.0979	55.0978
80	55.0891	55.0905	55.0917	55.0928	55.0938	55.0948	55.0956	55.0963	55.097	55.0976	55.098	55.0984	55.0987	55.0989	55.099	55.099
70	55.0891	55.0904	55.0916	55.0928	55.0938	55.0948	55.0956	55.0964	55.0971	55.0977	55.0982	55.0987	55.099	55.0993	55.0995	55.0996
60	55.0888	55.0901	55.0914	55.0925	55.0936	55.0946	55.0954	55.0962	55.097	55.0976	55.0981	55.0986	55.099	55.0993	55.0995	55.0996
50	55.0883	55.0897	55.0909	55.0921	55.0932	55.0942	55.0951	55.0959	55.0966	55.0972	55.0977	55.0982	55.0985	55.0988	55.0989	55.099
40	55.0875	55.089	55.0903	55.0915	55.0926	55.0936	55.0945	55.0952	55.0959	55.0965	55.097	55.0974	55.0977	55.0978	55.0979	55.0979
30	55.0865	55.088	55.0894	55.0906	55.0918	55.0928	55.0936	55.0944	55.0951	55.0956	55.096	55.0963	55.0964	55.0964	55.0964	55.0962
20	55.0853	55.0869	55.0883	55.0896	55.0908	55.0918	55.0926	55.0933	55.0939	55.0943	55.0946	55.0948	55.0948	55.0946	55.0943	55.0939
10	55.0838	55.0855	55.087	55.0884	55.0896	55.0906	55.0914	55.0921	55.0925	55.0928	55.093	55.0929	55.0927	55.0923	55.0917	55.091
0	55.0821	55.0839	55.0856	55.087	55.0882	55.0892	55.09	55.0905	55.0909	55.0911	55.091	55.0907	55.0902	55.0895	55.0886	55.0875
	-75	-65	-55	-45	-35	-25	-15	-5	5	15	25	35	45	55	65	75

X-Axis (mm)

Figure S15. The result of the curve fitting from y-axis to x-axis when the collar at location 4. From the results, the predicted location is at (75, 65). (Note: due to the values at (75, 60) and (75, 70) are the same, we chose the center of these two points as the predicted location).

Unit: mV

Y-Axis (mm)

100	55.0949	55.0957	55.0966	55.0976	55.0987	55.0999	55.1012	55.1026	55.1041	55.1057	55.1074	55.1093	55.1112	55.1132	55.1154	55.1176
90	55.0947	55.0955	55.0964	55.0973	55.0984	55.0995	55.1008	55.1021	55.1036	55.1051	55.1067	55.1084	55.1102	55.1121	55.1141	55.1162
80	55.0946	55.0953	55.0962	55.0971	55.0981	55.0992	55.1003	55.1016	55.1029	55.1044	55.1059	55.1075	55.1092	55.1109	55.1128	55.1147
70	55.0945	55.0952	55.096	55.0968	55.0977	55.0987	55.0998	55.101	55.1022	55.1035	55.1049	55.1064	55.108	55.1096	55.1114	55.1132
60	55.0946	55.0951	55.0958	55.0965	55.0974	55.0982	55.0992	55.1003	55.1014	55.1026	55.1039	55.1052	55.1067	55.1082	55.1098	55.1115
50	55.0947	55.0951	55.0956	55.0963	55.0969	55.0977	55.0986	55.0995	55.1005	55.1016	55.1027	55.104	55.1053	55.1067	55.1082	55.1097
40	55.0948	55.0951	55.0955	55.096	55.0965	55.0971	55.0978	55.0986	55.0995	55.1004	55.1015	55.1026	55.1038	55.105	55.1064	55.1078
30	55.0951	55.0952	55.0954	55.0957	55.096	55.0965	55.097	55.0977	55.0984	55.0992	55.1001	55.1011	55.1021	55.1033	55.1046	55.1059
20	55.0954	55.0953	55.0953	55.0953	55.0955	55.0958	55.0962	55.0966	55.0972	55.0979	55.0986	55.0995	55.1004	55.1015	55.1026	55.1038
10	55.0958	55.0954	55.0952	55.095	55.095	55.0951	55.0952	55.0955	55.0959	55.0964	55.097	55.0977	55.0986	55.0995	55.1005	55.1017
0	55.0962	55.0956	55.0951	55.0947	55.0944	55.0943	55.0942	55.0943	55.0945	55.0949	55.0953	55.0959	55.0966	55.0974	55.0984	55.0994
	-75	-65	-55	-45	-35	-25	-15	-5	5	15	25	35	45	55	65	75

X-Axis (mm)

Figure S16. The result of the curve fitting from x-axis to y-axis when the collar at location 5. From the results, the predicted location is at (75, 100).

Unit: mV

Y-Axis (mm)

100	55.0949	55.0957	55.0966	55.0976	55.0987	55.0999	55.1012	55.1026	55.1041	55.1057	55.1074	55.1093	55.1112	55.1132	55.1154	55.1176
90	55.0947	55.0955	55.0964	55.0973	55.0984	55.0995	55.1008	55.1021	55.1036	55.1051	55.1067	55.1084	55.1102	55.1121	55.1141	55.1162
80	55.0946	55.0953	55.0962	55.0971	55.0981	55.0992	55.1003	55.1016	55.1029	55.1044	55.1059	55.1075	55.1092	55.1109	55.1128	55.1147
70	55.0945	55.0952	55.096	55.0968	55.0977	55.0987	55.0998	55.101	55.1022	55.1035	55.1049	55.1064	55.108	55.1096	55.1114	55.1132
60	55.0946	55.0951	55.0958	55.0965	55.0974	55.0982	55.0992	55.1003	55.1014	55.1026	55.1039	55.1052	55.1067	55.1082	55.1098	55.1115
50	55.0947	55.0951	55.0956	55.0963	55.0969	55.0977	55.0986	55.0995	55.1005	55.1016	55.1027	55.104	55.1053	55.1067	55.1082	55.1097
40	55.0948	55.0951	55.0955	55.096	55.0965	55.0971	55.0978	55.0986	55.0995	55.1004	55.1015	55.1026	55.1038	55.105	55.1064	55.1078
30	55.0951	55.0952	55.0954	55.0957	55.096	55.0965	55.097	55.0977	55.0984	55.0992	55.1001	55.1011	55.1021	55.1033	55.1046	55.1059
20	55.0954	55.0953	55.0953	55.0953	55.0955	55.0958	55.0962	55.0966	55.0972	55.0979	55.0986	55.0995	55.1004	55.1015	55.1026	55.1038
10	55.0958	55.0954	55.0952	55.095	55.095	55.0951	55.0952	55.0955	55.0959	55.0964	55.097	55.0977	55.0986	55.0995	55.1005	55.1017
0	55.0962	55.0956	55.0951	55.0947	55.0944	55.0943	55.0942	55.0943	55.0945	55.0949	55.0953	55.0959	55.0966	55.0974	55.0984	55.0994
	-75	-65	-55	-45	-35	-25	-15	-5	5	15	25	35	45	55	65	75

X-Axis (mm)

Figure S17. The result of the curve fitting from y-axis to x-axis when the collar at location 5. From the results, the predicted location is at (75, 100).

4. The Raw Data of Figure 9 in the Paper

Because the values of voltage outputs in Figure 9 are not visible, we provide the raw data for each location in this supplementary material. The raw data of location E, L1, L2, R1, and R2 are shown in Figures S18–S22, respectively.

Unit: mV

Y-Axis (mm)																
100	40.776729	40.77457	40.77257	40.77074	40.76908	40.76758	40.76625	40.76509	40.76409	40.76326	40.7626	40.76211	40.76178	40.76161	40.76162	40.76179
90	40.775719	40.77385	40.77214	40.77059	40.7692	40.76796	40.76687	40.76595	40.76518	40.76456	40.76411	40.76381	40.76366	40.76368	40.76385	40.76417
80	40.775114	40.77357	40.77216	40.77089	40.76975	40.76876	40.76791	40.76719	40.76662	40.76618	40.76588	40.76572	40.7657	40.76582	40.76608	40.76648
70	40.774913	40.7737	40.77261	40.77162	40.77075	40.77	40.76936	40.76883	40.76841	40.76811	40.76793	40.76786	40.7679	40.76805	40.76832	40.76871
60	40.775118	40.77426	40.77349	40.7728	40.77219	40.77166	40.77121	40.77085	40.77056	40.77036	40.77024	40.7702	40.77024	40.77037	40.77057	40.77086
50	40.775728	40.77525	40.77481	40.77442	40.77407	40.77375	40.77349	40.77326	40.77307	40.77293	40.77282	40.77276	40.77274	40.77276	40.77283	40.77293
40	40.776742	40.77666	40.77657	40.77648	40.77638	40.77628	40.77617	40.77605	40.77593	40.7758	40.77567	40.77554	40.77539	40.77524	40.77509	40.77493
30	40.778162	40.7785	40.77877	40.77898	40.77914	40.77923	40.77926	40.77923	40.77915	40.779	40.77879	40.77852	40.7782	40.77781	40.77736	40.77685
20	40.779986	40.78076	40.7814	40.78193	40.78233	40.78261	40.78277	40.7828	40.78272	40.78251	40.78218	40.78173	40.78115	40.78045	40.77964	40.77869
10	40.782215	40.78344	40.78447	40.78531	40.78596	40.78642	40.78668	40.78676	40.78664	40.78633	40.78583	40.78514	40.78426	40.78319	40.78192	40.78046
0	40.78485	40.78655	40.78798	40.78914	40.79003	40.79066	40.79101	40.7911	40.79092	40.79047	40.78976	40.78877	40.78752	40.786	40.78421	40.78215
	-75	-65	-55	-45	-35	-25	-15	-5	5	15	25	35	45	55	65	75

X-Axis (mm)

Figure S18. The raw data of the location of E; the predicted location of collar is (−5, 0).

Unit: mV

Y-Axis (mm)																
100	40.768108	40.767534	40.76699	40.76649	40.766	40.76557	40.76517	40.76479	40.76445	40.76415	40.76387	40.76363	40.76343	40.76325	40.76311	40.76301
90	40.768608	40.768229	40.76786	40.76749	40.7671	40.76678	40.76644	40.7661	40.76577	40.76544	40.76513	40.76482	40.76452	40.76422	40.76393	40.76365
80	40.769032	40.768817	40.76859	40.76834	40.7681	40.7678	40.76751	40.76721	40.76688	40.76655	40.7662	40.76583	40.76545	40.76505	40.76464	40.76421
70	40.76938	40.769298	40.76918	40.76904	40.7689	40.76864	40.76839	40.76811	40.7678	40.76746	40.76708	40.76667	40.76622	40.76574	40.76523	40.76469
60	40.769651	40.769673	40.76965	40.76958	40.7695	40.76929	40.76908	40.76882	40.76852	40.76817	40.76777	40.76733	40.76683	40.7663	40.76571	40.76508
50	40.769846	40.769941	40.76998	40.76996	40.7699	40.76976	40.76958	40.76933	40.76904	40.76869	40.76828	40.76781	40.76729	40.76671	40.76608	40.76539
40	40.769965	40.770103	40.77018	40.77019	40.7701	40.77004	40.76988	40.76965	40.76936	40.76901	40.7686	40.76812	40.76759	40.76699	40.76634	40.76562
30	40.770007	40.770158	40.77025	40.77027	40.7702	40.77014	40.76998	40.76976	40.76948	40.76913	40.76873	40.76826	40.76773	40.76714	40.76648	40.76577
20	40.769974	40.770106	40.77018	40.7702	40.7702	40.77005	40.76989	40.76967	40.7694	40.76906	40.76867	40.76822	40.76771	40.76714	40.76652	40.76583
10	40.769863	40.769948	40.76998	40.76996	40.7699	40.76978	40.76961	40.76939	40.76912	40.7688	40.76843	40.76801	40.76753	40.76701	40.76644	40.76581
0	40.769677	40.769682	40.76965	40.76958	40.7695	40.76932	40.76913	40.76891	40.76864	40.76834	40.768	40.76762	40.7672	40.76674	40.76624	40.76571
	-75	-65	-55	-45	-35	-25	-15	-5	5	15	25	35	45	55	65	75

X-Axis (mm)

Figure S19. The raw data of the location of L1; the predicted location of collar is (−45, 30).

Unit: mV

Y-Axis (mm)

100	40.763128	40.76254	40.76194	40.76133	40.76071	40.76008	40.75944	40.7588	40.75814	40.75748	40.7568	40.75612	40.75543	40.75473	40.75402	40.7533
90	40.76291	40.7621	40.7613	40.7605	40.7597	40.7589	40.75811	40.75732	40.75654	40.75575	40.75497	40.7542	40.75343	40.75266	40.75189	40.75113
80	40.763034	40.76207	40.76112	40.76018	40.75927	40.75837	40.75749	40.75663	40.75579	40.75496	40.75416	40.75337	40.7526	40.75185	40.75111	40.7504
70	40.762868	40.7618	40.76077	40.75976	40.75878	40.75783	40.7569	40.75601	40.75514	40.7543	40.75349	40.7527	40.75195	40.75122	40.75052	40.74985
60	40.762412	40.76132	40.76025	40.75923	40.75823	40.75727	40.75634	40.75544	40.75458	40.75375	40.75296	40.7522	40.75147	40.75077	40.75011	40.74948
50	40.761666	40.7606	40.75958	40.75858	40.75762	40.7567	40.7558	40.75495	40.75412	40.75333	40.75257	40.75185	40.75116	40.75051	40.74988	40.7493
40	40.760629	40.75967	40.75873	40.75783	40.75695	40.75611	40.7553	40.75451	40.75375	40.75303	40.75233	40.75166	40.75103	40.75042	40.74984	40.74929
30	40.759302	40.7585	40.75772	40.75697	40.75623	40.75551	40.75481	40.75414	40.75348	40.75285	40.75223	40.75164	40.75107	40.75052	40.74998	40.74947
20	40.757685	40.75711	40.75655	40.75599	40.75544	40.7549	40.75436	40.75383	40.75331	40.75279	40.75228	40.75178	40.75128	40.75079	40.75031	40.74983
10	40.755777	40.7555	40.75521	40.75491	40.7546	40.75427	40.75393	40.75359	40.75323	40.75285	40.75247	40.75208	40.75167	40.75125	40.75082	40.75038
0	40.75358	40.75366	40.75371	40.75372	40.75369	40.75363	40.75354	40.75341	40.75324	40.75304	40.7528	40.75253	40.75223	40.75189	40.75151	40.7511
	-75	-65	-55	-45	-35	-25	-15	-5	5	15	25	35	45	55	65	75

X-Axis (mm)

Figure S20. The raw data of the location of L2; the predicted location of collar is (−75, 100).

Unit: mV

Y-Axis (mm)

100	40.756236	40.75691	40.75752	40.75807	40.75854	40.75895	40.7593	40.75957	40.75978	40.75992	40.76	40.76001	40.75995	40.75982	40.75963	40.75938
90	40.75708	40.75788	40.7586	40.75924	40.75979	40.76025	40.76063	40.76092	40.76113	40.76126	40.76129	40.76125	40.76112	40.7609	40.7606	40.76021
80	40.757669	40.75858	40.75939	40.76011	40.76072	40.76124	40.76165	40.76196	40.76218	40.7623	40.76231	40.76223	40.76204	40.76176	40.76138	40.7609
70	40.758002	40.759	40.7599	40.76068	40.76135	40.76191	40.76236	40.7627	40.76292	40.76304	40.76305	40.76295	40.76273	40.76241	40.76197	40.76143
60	40.758079	40.75915	40.76011	40.76095	40.76167	40.76227	40.76275	40.76312	40.76337	40.7635	40.76351	40.7634	40.76318	40.76284	40.76238	40.7618
50	40.757901	40.75903	40.76003	40.76091	40.76167	40.76231	40.76283	40.76323	40.7635	40.76366	40.76369	40.7636	40.76339	40.76306	40.7626	40.76203
40	40.757467	40.75863	40.75966	40.76058	40.76137	40.76205	40.7626	40.76303	40.76334	40.76353	40.76359	40.76354	40.76336	40.76306	40.76264	40.7621
30	40.756778	40.75795	40.75901	40.75995	40.76077	40.76147	40.76205	40.76252	40.76287	40.7631	40.76322	40.76321	40.76309	40.76285	40.7625	40.76202
20	40.755833	40.757	40.75806	40.75901	40.75985	40.76058	40.76119	40.7617	40.7621	40.76238	40.76256	40.76263	40.76258	40.76243	40.76216	40.76179
10	40.754633	40.75578	40.75683	40.75777	40.75862	40.75937	40.76002	40.76057	40.76102	40.76137	40.76163	40.76178	40.76184	40.76179	40.76165	40.7614
0	40.753177	40.75428	40.7553	40.75624	40.75709	40.75785	40.75853	40.75913	40.75964	40.76007	40.76042	40.76068	40.76085	40.76094	40.76095	40.76087
	-75	-65	-55	-45	-35	-25	-15	-5	5	15	25	35	45	55	65	75

X-Axis (mm)

Figure S21. The raw data of the location of R1; the predicted location of collar is (25, 50).

Unit: mV

Y-Axis (mm)																
100	40.758934	40.7587	40.75858	40.75857	40.75866	40.75887	40.75919	40.75961	40.76015	40.76079	40.76155	40.76241	40.76338	40.76447	40.76566	40.76696
90	40.758716	40.7587	40.75877	40.75893	40.75917	40.75951	40.75993	40.76045	40.76105	40.76174	40.76253	40.7634	40.76436	40.7654	40.76654	40.76777
80	40.758612	40.75876	40.75898	40.75927	40.75963	40.76007	40.76057	40.76115	40.76179	40.76251	40.7633	40.76416	40.76509	40.7661	40.76717	40.76832
70	40.75862	40.75889	40.75922	40.7596	40.76004	40.76054	40.76109	40.7617	40.76237	40.76309	40.76387	40.76471	40.7656	40.76655	40.76756	40.76862
60	40.758742	40.75909	40.75948	40.75992	40.7604	40.76093	40.7615	40.76212	40.76278	40.76349	40.76424	40.76504	40.76588	40.76677	40.7677	40.76867
50	40.758977	40.75936	40.75977	40.76023	40.76072	40.76124	40.7618	40.7624	40.76303	40.7637	40.76441	40.76515	40.76592	40.76674	40.76759	40.76847
40	40.759325	40.75969	40.76009	40.76052	40.76098	40.76147	40.76199	40.76254	40.76312	40.76373	40.76437	40.76504	40.76574	40.76647	40.76723	40.76802
30	40.759787	40.7601	40.76044	40.7608	40.7612	40.76162	40.76206	40.76254	40.76304	40.76357	40.76413	40.76471	40.76532	40.76596	40.76663	40.76732
20	40.760361	40.76057	40.76081	40.76107	40.76136	40.76168	40.76203	40.7624	40.7628	40.76323	40.76368	40.76416	40.76467	40.76521	40.76577	40.76637
10	40.761049	40.76111	40.7612	40.76133	40.76148	40.76166	40.76188	40.76212	40.76239	40.7627	40.76303	40.7634	40.76379	40.76422	40.76468	40.76516
0	40.76185	40.76172	40.76163	40.76157	40.76155	40.76156	40.76161	40.7617	40.76183	40.76199	40.76218	40.76242	40.76268	40.76299	40.76333	40.76371
	-75	-65	-55	-45	-35	-25	-15	-5	5	15	25	35	45	55	65	75

X-Axis (mm)

Figure S22. The raw data of the location of R2; the predicted location of collar is (75, 60).

5. The Evaluation of Minimum Bronchus Which the Probe with Collar is able to Access

To evaluate the bronchus which allows our collar to travel, we survey some lecture for diameters of different bronchus. Researchers reported that diameters of a primary bronchi, secondary bronchi, tertiary bronchi, and bronchioles are about 12.2, 8.3, 5.6, and 4.5 mm, respectively [1]. We summarize the diameter of different bronchus in Table S1. According to their study, the collar in this paper only can reach the secondary bronchi. To reduce the diameter of collar while still remaining the same sensing performance, we are currently studying two methods. The first one method is to investigate different materials owning better sensing response and consequently use the materials to make smaller collars (i.e., to replace the current large silicon-steel collar). We use a nickel sheet and a permalloy (Ni-Fe) sheet to make smaller collars. These sheets and the silicon-steel sheet are compared together by conducting a test to evaluate how these materials influence the voltage output of the receiving electromagnet. As shown in Figure S23, the test setup includes one pair of electromagnets and corresponding measurement electronics (for testing, we only change the materials while other parameters are fixed). Before the testing, we apply ac current with a frequency of 500 Hz to the emitting electromagnet, and subsequently measure the voltage output of the receiving electromagnet by using a lock-in amplifier. This voltage output is used as a background voltage output. After the background voltage is obtained, we put these sheets in the gap between the emitting electromagnet and the receiving electromagnet. After this, we measure the voltage output again and analyze/record the change of voltage output. We use the value of change of voltage output to further calculate change of voltage output per unit volume of material usage. The results are summarized in Table S2. The Table S2 shows that the voltage change per unit volume of material usage is largest for the permalloy sheet (and is about 2.5 time larger than that for silicon-steel sheet). This indicate that we can minimize the diameter of the collar by replacing the silicon steel sheets to permalloy sheets, while still can remaining the same sensing performance.

The second method is to optimize the receiving electromagnets to get a better sensitivity by using theoretical analysis [2]. By optimization, the receiving electromagnets are able to detect smaller changes of magnetic flux which are induced by smaller collars, as we mention in our paper. Due to this, even though the material selection/usage of collar (above paragraph) has reached a limitation of sensitivity increasing, we still can continuously minimize the collar by optimization of the receiving electromagnets. Moreover, if the optimization can be conducted for the receiving electromagnets, emitting coil, and associate parts (from system perspectives), we believe the collar will be significantly miniaturized and thus can reach most bronchioles.

Table S1. Typical Diameter of Bronchus.

Type of Bronchus	Outer Diameter of Bronchus
Primary Bronchi	12.2 mm
Secondary Bronchi	8.3 mm
Tertiary Bronchi	5.6 mm
Bronchioles	4.5 mm

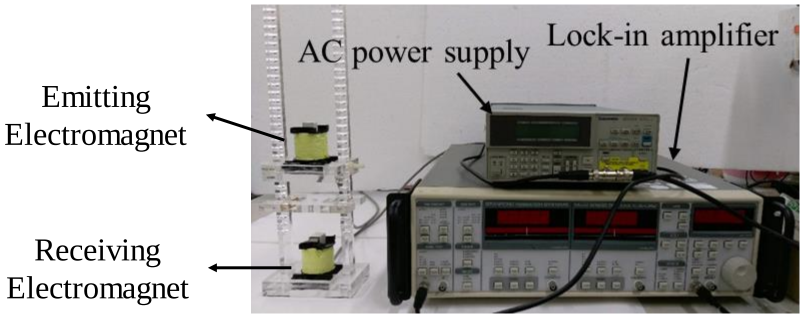

Figure S23. The test setup for sensing performance by using different materials for the collar.

Table S2. The Summary of the change of voltage output of different materials.

Material	Nickel	Permalloy (80% Ni, 5% Mo, 15% Fe)	Silicon-Steel
Volume (mm^3)	10 × 10 × 0.1 = 10	10 × 10 × 0.1= 10	10 × 10 × 0.23 = 23
Background voltage output (mV)	68.33	68.86	68.77
Change of voltage output (μV)	33.62	300.32	277.99
Change of Voltage Output per Unit Volume of Material Usage (μV/mm^3)	3.362	30.032	12.087

6. Methods to Improve Our System for Locating the Collar More Accurately

In following, we provide some methods to improve our system for locating the collar more accurately. The first method is increasing diameter of receiving electromagnets. Recently, some researchers reported a theoretical analysis electromagnet-based sensor [2] to estimate the voltage output of an electromagnets. In their paper, the voltage output of an electromagnet-based sensor is below.

$$\mathrm{V} = 0.9 \times 10^{-5} \times f \times \frac{l^3}{d^2} \times D_i \times \frac{1}{\ln\left(2\frac{l}{D_i}\right) - 1} H$$

Where the f is frequency of magnetic field, l is the length of the electromagnet, d is diameter of winding wire, D_i is the diameter of the ferromagnetic core in the electromagnet. The paper also indicates the optimal diameter of ferromagnetic core is 30% of outer diameter of electromagnets, D. Thus, we assume that the ratio of core diameter and electromagnet diameter are unchanged, the equation can be written as

$$\mathrm{V} = 0.9 \times 10^{-5} \times f \times \frac{l^3}{d^2} \times 0.3D \times \frac{1}{\ln\left(2\frac{l}{0.3D}\right) - 1} H$$

According to this equation, if the other parameters in above equation are fixed, when the diameter increases, the voltage output also increases. According to this relation, if a small change of magnetic flux induced by the small movement of the collar, the electromagnet with larger diameter is able to convert the small change of magnetic flux into a large change of voltage output. Due to this, the small movement of collar is able to be detected by system and thus the accuracy can be increased.

Second method is increasing the number of electromagnets within the same planar area of the electromagnets-array (i.e., increasing the sensing-resolution). When the amount of receiving electromagnets is increased in the same area, the pitches between each electromagnet in x-axis and y-axis are decreased (i.e., the resolution of sensing are increased). Due to this, the system can record more experimental results along x-axis and y-axis in the same area and consequently use more experimental results to conduct the more accurate regression analysis. More accurate regression analysis can have better analyzed/predicted results to match the experimental results more precisely. That is, the predicted location of the collar can be closer to the actual location of the collar. Hence, the accuracy is increased. According to above reason, either increasing the diameter or number of receiving electromagnets will increase the accuracy.

However, when we increase the diameter of electromagnets, the electromagnets with larger diameters occupy more footprint within the original area of the electromagnet-array (thus x- and y-gaps between the outer-periphery of each electromagnet in the electromagnets-array are decreased). Therefore, when the x- and y- gaps are decreased, the induction-interferences between electromagnets are increased. Moreover, when the diameter of each electromagnet is continuously increased, eventually the number of electromagnets must be decreased in order to be arranged/fitted within the original same area of the electromagnets-array. In this case, decreasing the number of

electromagnets will worsen the targeting resolution (i.e., the targeting system will record less experimental results within the same area). When less experimental results are imported to the signal-process program to conduct curve fitting, the fitting curve will be less accurate and thus the targeting accuracy will be decreased. Due to this reason, we cannot always increase the diameter of the electromagnets. Instead, we have to consider the trade-off issue between the diameter and the number of electromagnets. In addition to above two parameters, it is better to investigate the other parameters influencing the targeting accuracy together at the same time (i.e., considering not only the receiving electromagnets, but also emitting electromagnets, etc) and consequently optimize these parameters from system perspectives. However, optimization of the receiving electromagnets-array (or optimization of the receiving and emitting electromagnets-arrays) from system perspective require a long-term effort. Thus, we do not conduct the optimal design in this manuscript, but we still add above paragraphs describing the influence caused by the diameter and the number of the receiving electromagnets to the targeting accuracy.

The third method is to improve the regression analysis to increases the accuracy. We can use other functions, such as Gauss distribution or higher order polynomial function, to conduct the regression analysis. Due to this, the regression analysis may match the experimental results more accurately. Moreover, in our system, we use 16 points and 11 points to plot the fitting curve along x and y axis, respectively. However, this causes that the fitting curve is not smoothly plotted. Due to this, the actual local-largest-point would not be calculated/plotted in the fitting curve. Instead, we may obtain the second local-largest-point which is just close to the actual local-largest-point. However, when we use more points for calculation, we can plot more points to obtain more smooth-fitting curves. Thus, second local-largest-point may approach more closely to the actual local-largest-point, even when the actual local-largest-point still cannot be calculated and plotted. Due to this, the accuracy is increased. However, when the number of points is increased, the calculation time of signal-processing program is also increased. This results in a time-delay of the targeting. Even though this delay issue can be addressed by improving the signal-processing program, the optimizing signal-processing is not the main research topic in this paper. Due to above reason, we only use 16 points in x-axis at this moment and will optimize the signal-processing program as future work.

Reference:

1. Michael, G.L.; Chapter 1. Function and Structure of the Respiratory System, In *Pulmonary Physiology*, 8th ed.; McGraw-Hill Companies: New York, NY, USA 2013.
2. Tumanski, S. Induction coil sensors—a review, *Meas. Sci. Technol.* **2007**, *18*, R31-R46.

© 2019 by the authors. Licensee MDPI, Basel, Switzerland. This article is an open access article distributed under the terms and conditions of the Creative Commons Attribution (CC BY) license (http://creativecommons.org/licenses/by/4.0/).
